# Functional plasticity of glutamatergic neurons of medullary reticular nuclei after spinal cord injury in mice

**DOI:** 10.1038/s41467-024-45300-4

**Published:** 2024-02-20

**Authors:** Maxime Lemieux, Narges Karimi, Frederic Bretzner

**Affiliations:** 1 Centre de Recherche du CHU de Québec, CHUL-Neurosciences, 2705 Boul. Laurier, Québec, QC G1V 4G2 Canada; 2 Faculty of Medicine, Department of Psychiatry and Neurosciences, Université Laval, Québec, QC G1V 4G2 Canada

**Keywords:** Motor control, Neurology, Spinal cord diseases

## Abstract

Spinal cord injury disrupts the descending command from the brain and causes a range of motor deficits. Here, we use optogenetic tools to investigate the functional plasticity of the glutamatergic reticulospinal drive of the medullary reticular formation after a lateral thoracic hemisection in female mice. Sites evoking stronger excitatory descending drive in intact conditions are the most impaired after injury, whereas those associated with a weaker drive are potentiated. After lesion, pro- and anti-locomotor activities (that is, initiation/acceleration versus stop/deceleration) are overall preserved. Activating the descending reticulospinal drive improves stepping ability on a flat surface of chronically impaired injured mice, and its priming enhances recovery of skilled locomotion on a horizontal ladder. This study highlights the resilience and capacity for reorganization of the glutamatergic reticulospinal command after injury, along with its suitability as a therapeutical target to promote functional recovery.

## Abstract (web summary)

Spinal cord injury disrupts the descending command from the brain necessary for locomotion. Here, the authors show the functional plasticity of glutamatergic reticulospinal neurons and how their recruitment can enhance spontaneous motor recovery.

## Introduction

Spinal cord injury (SCI) is a neurotraumatic disorder affecting locomotor gait and posture. Most SCIs are incomplete and spare some descending supraspinal pathways of the brain^1^, thus opening the possibility of using neuro-prosthetic approaches to investigate their contribution to spontaneous motor recovery and to test whether their stimulation can improve functional outcomes. While there is an abundant literature on the functional role of the motor cortex and its corticospinal pathways after SCI^2–5^, less is known about reticulospinal pathways of the medullary reticular formation (MRF), their plasticity, and possible involvement in spontaneous motor recovery from SCI.

The MRF includes mainly the gigantocellular reticular nucleus (Gi), the alpha and ventral parts of the Gi (GiV/α), and the lateral paragigantocellular nucleus (LPGi)^6^. Optogenetic activation of glutamatergic neurons of the Gi modulates locomotor pattern in intact mice^7^. Some glutamatergic neurons of the Gi have been genetically identified by their LHX3/CHX10 transcription factor expression as V2a neurons^8^, and this subpopulation is involved in several different motor functions: their bilateral stimulation can induce locomotor stop^9^, whereas their unilateral stimulation evokes head turning and body steering^10,11^.

After SCI, the density of reticulospinal axonal terminals of unidentified Gi neurons increases onto propriospinal interneurons located above and below the lesion site^12–14^. Pharmacogenetic silencing of reticulospinal neurons of the Gi highlights its contribution to spontaneous locomotor recovery from SCI^15^. Although pharmacogenetic silencing of glutamatergic neurons of the GiV has no effects on locomotion in intact mice, its inhibition precludes locomotor recovery of chronic SCI mice promoted by cortical stimulation^5^. These studies suggest that the Gi and GiV/α contribute to locomotor recovery after SCI.

Using brainstem-spinal cord preparations isolated from neonatal rodents, electrical or chemical stimulation in the vicinity of the LPGi or optogenetic activation of glutamatergic neurons of the LPGi have been shown to induce locomotor-like activity^16–18^. Moreover, photoactivation of glutamatergic neurons of the LPGi can also induce locomotion and accelerate locomotor speed of adult mice in normal conditions^19^, presumably by relaying mesencephalic locomotor region inputs^20,21^, thus suggesting that glutamatergic neurons of the LPGi could contribute to recovery of locomotor rhythm after SCI.

In this study, we evaluated the functional plasticity of glutamatergic neurons of the Gi, GiV/α, LPGi, and the intermediate reticular nucleus (IRn) to spontaneous motor recovery after SCI and tested whether their stimulation could boost functional outcomes after chronic SCI. Combining detailed kinematics and electromyographic recordings in freely behaving mice with optogenetic manipulation, we show that functional plasticity of reticulospinal efficacy of glutamatergic neurons of MRF nuclei occurs concurrently with spontaneous motor recovery from SCI. Functionally, if photostimulation of glutamatergic neurons located in the LPGI and GiV/α initiated and accelerated locomotion after SCI, glutamatergic neurons located in the Gi stopped locomotion. As a potential therapeutical approach, we also show that tonic activation of glutamatergic neurons of the MRF for priming the descending drive improved skilled locomotor control after chronic SCI.

## Results

### Partial locomotor recovery following a lateral thoracic hemisection

**Fig. 1:**
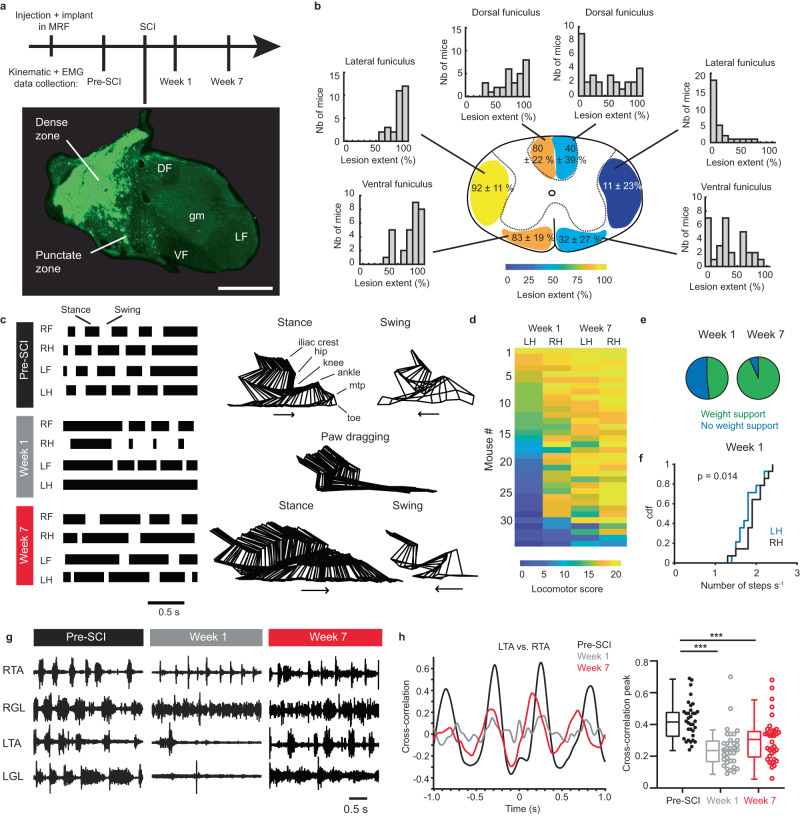
Impact of hemisection on hindlimb locomotion. **a** Experimental timecourse and example of a thoracic section illustrating the lesion. The lesion is autofluorescent. Scale bar, 0.5 mm. DF dorsal funiculus, gm gray matter, LF lateral funiculus, VF ventral funiculus. **b** Extent of lesioned white matter on the right and left side (*n* = 34 mice). **c** Locomotor impairment caused by SCI shown with gait and stick diagrams before and after SCI. LF left forelimb, LH left hindlimb, RF right forelimb, RH right hindlimb. **d** Non-linear locomotor score at week 1 and 7 for the left (LH) and right (RH) hindlimb. **e** Pie charts of the fraction of mice with (green) and without (blue) weight support after SCI. **f** Cumulative distribution function of the step frequency of left and right hindlimbs at weeks 1 and 7. Two-tailed paired *t*-test, *t*_(13)_ = 2.83, *p* = 0.0142. **g** EMG recordings before and after SCI for the ankle flexor Tibialis anterior (RTA and LTA) and ankle extensor/knee flexor Gastrocnemius lateralis (RGL and LGL). **h** Cross-correlograms of the LTA vs. RTA. Right, boxplot of cross-correlation peaks (*n* = 34 mice). Center of the boxplot is the median, upper and lower limits of the box are the first and third quartile respectively and the upper and lower limits of whiskers are the maxima and minima contained within the Tukey’s distance. One-way repeated ANOVA, *F*_(2,66)_ = 20.30, *p* < 0.0001, with post hoc Tukey HSD tests: pre-SCI vs. week 1, ***p < 0.0001; pre-SCI vs. week 7, ***p = 0.0002; week 1 vs. week 7, *p* = 0.118. Source data are provided as a Source Data file.

We used a lateral thoracic hemisection as a model to investigate changes in the descending reticulospinal drive to the ipsilesional lumbar network after SCI. The lesion disrupted most of the left spinal cord with a dense zone of fibrotic tissue surrounded by a punctate zone reminiscent of swollen axons (Fig. 1a), whereas most of the right side was spared (Fig. 1b, c). On the left side (ipsilesional side), the lesion extent was maximal in the lateral funiculi and severed about 80% of the ventral and dorsal funiculi (coefficient of variation 12–27%). On the right side, the lesion was more variable (coefficient of variation of 80–200%), usually extending on average to about 32% of the ventral funiculus and 40% of the dorsal funiculus, but it avoided most of the lateral funiculus (Fig. 1b). Statistically, the lesion extent was similar for funiculi on the same side and different from those on the other side (Supplementary Fig. 1a–c). Mice were evaluated after 1 week to determine the impairment at the beginning of the subacute phase and after 7 weeks to evaluate the level of recovery in the intermediate/early chronic phase^22^. Behaviorally, the ipsilesional hindlimb exhibited variable locomotor deficits (Fig. 1c, d and Supplementary Fig. 1d) ranging from paresis, including a loss of body weight support and a paw dragging (Fig. 1e), to fewer steps of the ipsilesional hindlimb than the contralesional one a week after SCI (Fig. 1f).

To further detail locomotor deficits, electromyographic (EMG) activity was recorded. EMG activity was largely decreased 1 week after SCI in both ipsilesional flexor and extensor muscles (Fig. 1h, left Tibialis anterior or LTA and Gastrocnemius lateralis or LGL). Regardless of their recovery over time, both locomotor pattern and rhythm remained less coordinated and more irregular than before injury (Fig. 1h). The level of residual impairment of both hindlimbs 7 weeks after SCI could be predicted by the extent of lesion, especially in both lateral funiculi (Supplementary Table 1).

### No lateralization of reticulospinal efficacy after SCI

**Fig. 2:**
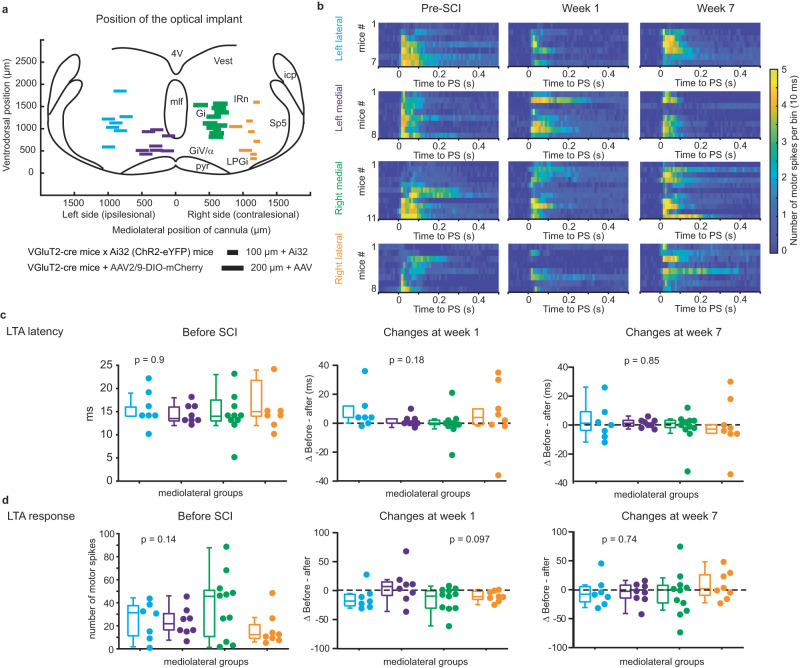
No effect of lateralization on changes caused by SCI at rest. **a** Position of the optical implant evaluated from histological sections of each mouse with a color-coding for each group: left lateral (blue, *n* = 7), left medial (purple, *n* = 8), right medial (green, *n* = 11), and right lateral (orange, *n* = 8). 4V fourth ventricle, Gi gigantocellular nucleus, GiV/α gigantocellular pars ventral and pars alpha, icp inferior cerebellar peduncle, IRn intermediate reticular nucleus, LPGi lateral paragigantocellular nucleus, mlf medial longitudinal fasciculus, pyr pyramidal tract, Sp5 spinal trigeminal nucleus. **b** Color-coded matrix of the LTA response to pulse of 10 ms at rest (suprathreshold intensity). Each row is an averaged number of LTA motor spikes per 10 ms bins (10–20 trials). Lines are identified on the right for VGluT2-cre::Ai32 transgenic mice. **c** Distribution of latencies of the LTA (left) and changes at week 1 (middle) and week 7 (right). Left lateral, *n* = 7; left medial, *n* = 8, right medial, *n* = 11, and right lateral, *n* = 8. Before SCI, two-tailed Kruskal–Wallis test, *χ*^2^ = 0.618, *p* = 0.89; changes at week 1, Kruskal–Wallis test, *H*_(3)_ = 4.471, *p* = 0.21; changes at week 7, Kruskal–Wallis test, *χ*^2^ = 1.211, *p* = 0.75. **d** Distribution of EMG response of the LTA (left) and changes at week 1 (middle) and week 7 (right). Before SCI, one-way ANOVA test, *F*_(3,30)_ = 1.927, *p* = 0.14; changes at week 1, two-tailed one-way ANOVA, *F*_(3,30)_ = 2.877, *p* = 0.0525; changes at week 7, one-way ANOVA test, *F*_(3,30)_ = 0.4604, *p* = 0.712. Center of boxplots is the median, upper and lower limits of boxes are the first and third quartile respectively and the upper and lower limits of whiskers are the maxima and minima contained within the Tukey’s distance. Source data are provided as a Source Data file and on Figshare^47^ as the dataset “EMG responses to photostimulation at rest” (10.6084/m9.figshare.c.6925099.v1).

We hypothesized that a lateral hemisection would impair the descending drive ipsilateral to the lesion, while sparing the contralateral one. Using Vglut2-cre transgenic mice, we drove expression of the photoactivator Channelrhodopsin-2 in glutamatergic neurons and implanted an optical fiber to activate a spatially restricted (100–200 µm) region within the ipsilesional (*n* = 15 mice) or contralesional (*n* = 19 mice) MRF (Fig. 2a and Supplementary Fig. 2). We used the pyramidal tract on coronal sections as an anatomical landmark to delineate stimulation sites from the medial (Gi and GiV/α) vs. lateral MRF (LPGi and IRn). Using EMG recordings of the ipsilesional LTA in mice at rest, we measured latency and the number of motor spikes (as a proxy of motor efficacy) evoked upon 10 ms photostimulation pulses (Fig. 2b). These motor responses were evoked within 10–25 ms (median 14 ms) before lesion (Fig. 2c). The latency was slightly longer at week 1 after SCI (median 18 ms, Wilcoxon signed rank test, *p* = 0.044) but returned to pre-SCI levels at week 7 (median 16 ms, Wilcoxon signed rank test, *p* = 0.92). Although independent of the stimulation site (Fig. 2c), changes in latency were also affected by the lesion size (Supplementary Table 1). Unexpectedly, changes in motor responses were independent of the stimulation site and lesion size over time after SCI (Fig. 2d), thus suggesting that the descending glutamatergic reticulospinal drive kept access to the lumbar spinal circuit below the lesion.

### Changes in reticulospinal efficacy during locomotion after SCI

**Fig. 3:**
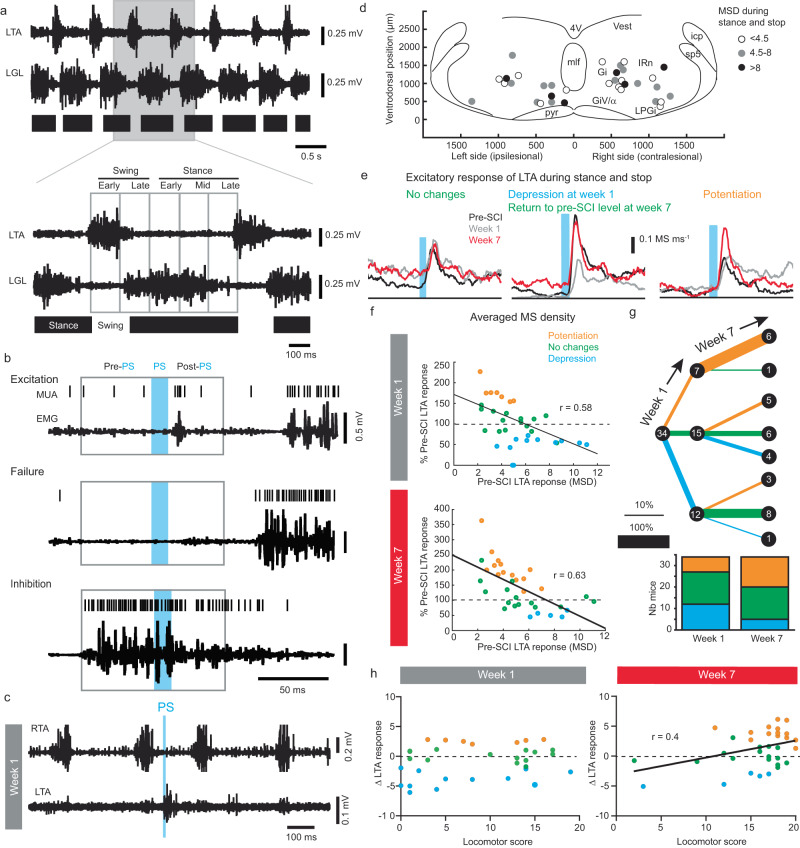
Changes after SCI of LTA response to MRF inputs during locomotion. **a** Examples of EMG recordings of the left tibialis anterior (LTA) and gastrocnemius lateralis (LGL) along a gait diagram. Region highlighted in gray is enlarged below to illustrate the division of the step cycle in 5 bins. **b** Three examples of EMG recordings of the LTA during a period of photostimulation (PS, period in blue). Boxes indicate time windows for motor spike density (MSD) computation. **c** Example of the LTA response at week 1 while the left limb is dragging. **d** Schematic of the location of the optical fiber grayscale-coded according to the MSD excitatory response (*n* = 34 mice). Groups of MSD magnitudes were determined by k-means. **e** Averaged MSD excitatory response showing the different types of changes: no changes (green), depression (blue, here with recovery in green), and potentiation (orange). **f** Scatter plots of changes in response (% pre-SCI) at week 1 and 7 of the MSD vs. averaged pre-SCI response. Averaged response per mouse (each circle) were obtained from 36 ± 13 trials pre-SCI, 35 ± 16 trials at week 1, and 28 ± 17 trials at week 7. Two-sided simple linear regression: at week 1, *F*_(1,32)_ = 17.72, *p* = 0.0002, *R*^2^ = 0.336; at week 7, *F*_(1,32)_ = 22.54, *p* < 0.0001, *R*^2^ = 0.395. **g** Graphs illustrating the proportion of mice in each case at week 1 and further changes from week 1 to week 7. Bar graphs at the bottom indicate proportions of each case for the whole sample. **h** Scatter plot of the absolute change of MSD response after SCI vs. locomotor score. Two-sided simple linear regression: at week 1, *F*_(1,32)_ = 1.982, *p* = 0.169; at week 7, *F*_(1,32)_ = 6.119, *p* = 0.0189, *R*^2^ = 0.16. Source data are provided as a Source Data file and on Figshare^47^ as the dataset “Data and script for EMG response during locomotion (10 ms)” (10.6084/m9.figshare.c.6925099.v1).

Given the importance and necessity of several neuronal populations of the MRF to spontaneous motor recovery after SCI^5,15^, we hypothesized that the glutamatergic reticulospinal drive should retain some of its efficacy after SCI. To investigate this, we assessed changes over time in reticulospinal efficacy on the ipsilesional LTA during treadmill locomotion at steady speed before and after SCI (Fig. 3). As the descending reticulospinal drive depends on the state of the step cycle^7^, the step cycle was divided in 5 equal bins starting with LTA burst onset (Fig. 3a and Supplementary Fig. 3a). The number of motor spikes was evaluated in 50 ms time windows (referred to as motor spikes density or MSD) upon 10 ms photostimulation pulses. Motor responses were defined as the difference of MSD post- vs. pre-photostimulation (background activity in Supplementary Fig. 3b). Three types of response were identified: excitation, inhibition, or failure (Fig. 3b and Supplementary Fig. 3c–e). Excitation was the dominant response to photostimulation of the MRF before and after SCI (Supplementary Fig. 3c). Inhibition was less common than excitation and was observed mainly during late swing when the burst normally terminates before SCI and at week 7. It was more frequent throughout the step cycle during week 1 (Supplementary Fig. 3d). Also of lower occurrence, failure was observed mainly during the stance phase and remained low after SCI (Supplementary Fig. 3e).

Because it was not possible to distinguish spontaneous motor activity from motor responses during its activated phase (swing) and the late stance, we focused on the unambiguous motor responses evoked during the relaxed phases of the muscle (early and mid-stance as well as stops) because the locomotor background activity was minimal (Supplementary Fig. 3a, b). Given that motor responses were similar during stance and stops after SCI (Supplementary Fig. 3f), data were pooled for the analysis of plasticity during locomotion (Fig. 3e–h). Using each mouse as its control, we evaluated the direction of plasticity (no changes, depression, or potentiation) after SCI (Fig. 3e, Mann–Whitney rank sum test). There was no effect of lateralization on the proportion of excitation, inhibition, and failure rate or on the response efficacy for all investigated time points (Fig. 3d and Supplementary Fig. 4). Instead, we found an anti-correlation of changes in excitatory motor responses vs. pre-SCI responses, with sites evoking the strongest motor responses pre-SCI ending up depressed while those evoking the weakest responses getting potentiated (Fig. 3f).

Regarding the time course of plasticity (Fig. 3g), 15 of the 34 stimulated sites did not show any changes in excitatory motor responses at week 1 after SCI; nevertheless, their responses were either depressed or potentiated at week 7 after SCI in almost two-thirds of these mice (9/15). Depression was the next most common observation (12/34) at week 1, but either returned to pre-injury levels or was potentiated at week 7 after SCI. While there was no correlation between plasticity and locomotor score at week 1, correlation was observed at week 7 (Fig. 3h).

Because the response duration was longer at rest than during locomotion (Supplementary Fig. 5a), presumably through the recurrent activity of the spinal circuit, we expected a larger depression at rest than during locomotion. However, similar levels of depression were found at week 1 (14/34 at rest vs. 12/34 during locomotion, Fisher’s exact test, *p* = 0.803) and week 7 after SCI (10/34 at rest vs. 5/34 during locomotion, Fisher’s exact test, *p* = 0.242; Supplementary Fig. 5b, c). Furthermore, the anti-correlation between pre-lesion levels and changes in excitatory response observed during locomotion was also observed at rest (Supplementary Fig. 5d). While the extent of the lesion size had no influence on the plasticity of motor responses at rest, it influenced excitatory motor responses during locomotion. Indeed, the most extensive lesion on the left side promoted potentiation, whereas the largest lesion on the right side led to depression (Supplementary Table 1).

Considering the variation in motor spike density response as a correlate of recruitment, we next investigated the variation of motor spike amplitude to evaluate the synchronization of motor pools. Before SCI, the amplitude of motor spikes was largest during the active phase of the LTA burst and smallest during the relaxed phase, with the amplitude of motor responses ranging between these minima and maxima. Disrupted at week 1, the phase-dependent modulation of motor spike amplitude returned to pre-injury level by week 7 (Supplementary Fig. 6a–c). Anti-correlation of changes and pre-SCI levels observed for motor spike density was also observed for the amplitude (Supplementary Fig. 6d), and the time-course of plasticity was similar overall (Supplementary Fig. 6e). Finally, amplitude and density were correlated at all time points (Supplementary Fig. 6f), suggesting that recruitment and synchronization of the LTA motor pool by the descending glutamatergic drive were similarly modulated.

In summary, changes in motor responses during locomotion and longer responses at rest occurred regardless of the stimulation site. Nevertheless, changes over time in reticulospinal efficacy following SCI occur alongside spontaneous motor recovery after SCI.

### MRF initiates, modulates, or stops walk after chronic SCI

**Fig. 4:**
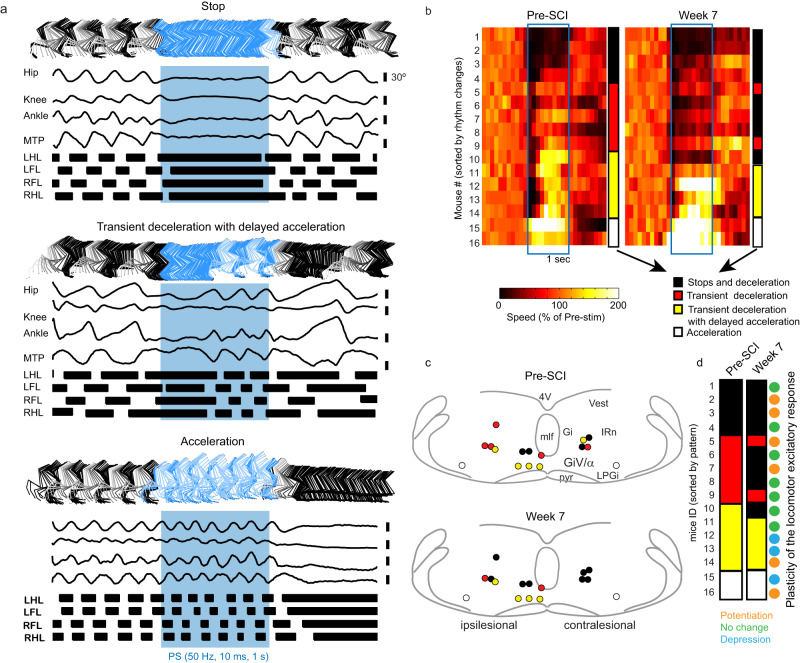
Effect of trains of photostimulation of medullary reticular formation on locomotor activity. **a** Stick diagrams, angular excursion of the hip, knee, ankle, and metatarsophalangeal (MTP) joints; and gait diagrams during locomotor stops/resets, transient deceleration with delayed acceleration and during acceleration. **b** Color-coded matrices of averaged locomotor speed (*n* = 4–7 trials per mouse). Lines (*n* = 16 mice) were sorted according to the effect on speed, ranging from full stop (black) to acceleration (white). In between, there were transient decelerations (red) and deceleration followed by delayed acceleration (yellow). Color coding is used in subsequent (**c**) and (**d**). **c** Locations of sites color-coded according to the effect seen in (**c**). 4V fourth ventricle, Gi gigantocellular nucleus, Giα/GiV gigantocellular pars alpha and pars ventral, IRn intermediate reticular nucleus, LPGi lateral paragigantocellular nucleus, mlf medial longitudinal fasciculus, pyr pyramidal tract. **d** Schematic of the effect of trains of photostimulation before and at week 7 after SCI, aligned with plasticity of excitatory response as shown in Fig. 3. Source data are provided as a Source Data file.

Using a subset of mice (Fig. 4, *n* = 16 mice), we next evaluated whether a train of photostimulation at 50 Hz for 0.5–1 s delivered above glutamatergic neurons of contra- or ipsilesional MRF sites changes locomotor pattern and rhythm before and 7 weeks after SCI. Whereas some MRF stimulation sites elicited locomotor stops, others promoted acceleration before and after SCI (Fig. 4a, b). In between these two opposite effects, most sites showed a transient deceleration that was sometimes followed by a delayed acceleration (as illustrated by red and yellow bars in Fig. 4b).

Regarding the location of stimulation sites, most sites evoking a locomotor stop (Fig. 4c, black circles) were located in the Gi nucleus. In contrast, sites evoking acceleration (Fig. 4c, white circles) were located in the region of the LPGi, whereas those evoking a delayed acceleration (Fig. 4c, yellow circles) were located mainly in the vicinity of the GiV/α. The last sites evoking a transient deceleration (red bars) were generally located dorsally and more laterally, corresponding to the lateral part of the Gi and the IRn. These results are in line with previous studies^7,9–11,19^. The ability to promote acceleration was preserved after chronic SCI, while transient deceleration disappeared and gave rise to a higher occurrence of locomotor stops (Fig. 4b). These suggest that SCI induced plasticity of the descending reticulospinal drive of sites to promote stops, but spared sites that promote acceleration. However, these changes in dynamics triggered by 1-s 50 Hz trains were not predicted by the lateralization of the stimulation site (i.e., ipsi- vs. contralesional side, Fig. 4c), nor by the plasticity of the locomotor response to shorter (10 ms) pulses (Fig. 4d).

### MRF modulates ipsilesional hindlimb motoneuronal activity after SCI

**Fig. 5:**
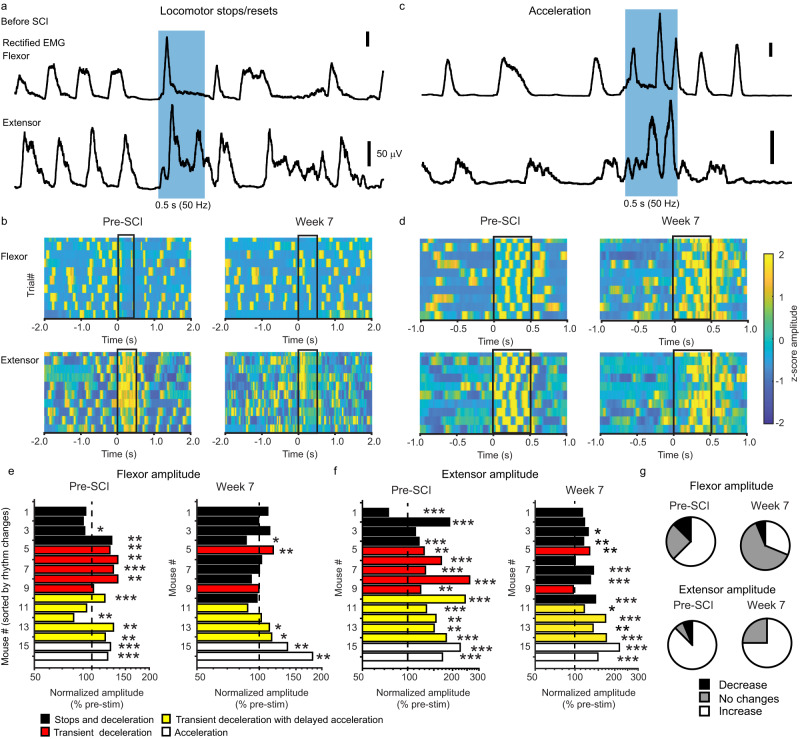
Changes after SCI in the recruitment of flexors and extensors during trains of photostimulation. Examples of rectified EMG activity of flexors (Tibialis anterior) and extensor (Gastrocnemius lateral) shown as rectified EMG for a single example (**a**, **c**) and color-coded matrices for 10 trials (**b**, **d**) during stops (**a**, **b**) and acceleration (**c**, **d**). **e**, **f** Bar graph (sorted and color-coded as in Fig. 4, *n* = 16 mice) of changes in amplitude (averaged over 10–25 trials) caused by photostimulation (normalized by pre-stimulation amplitude) before and 7 weeks after SCI of the flexor (**e**) and extensor (**f**). Stops and deceleration in black, transient deceleration in red, transient deceleration with delayed acceleration in yellow and acceleration in white. Significance of photostimulation effect was tested with a two-sided Wilcoxon signed rank test, *p value < 0.05, **p value < 0.01. Exact *p* values are provided in Source data. **g** Pie charts of the effects of MRF photostimulation on EMG amplitude. Decrease in black, no changes in gray and increase in white. Source data are provided as a Source Data file and on Figshare^47^ as the dataset “Data and script for EMG response during locomotion (500 ms)” (10.6084/m9.figshare.c.6925099.v1).

We next assessed whether a photostimulation train modulates propulsion during acceleration and slows down during deceleration/stop before and after SCI. To test this, we monitored EMG locomotor activity as a proxy of muscle recruitment under the premise that an increase in amplitude could reflect the synchronization of relevant flexor or extensor motoneuronal pools (Fig. 5). Sites evoking locomotor stop or reset induced an initial flexor burst along with a sustained increase in extensor activity, thus leading to an initial flexor/extensor co-activation essential for slowing down and an increased duration in extensor muscles necessary for braking locomotor activity before SCI. Flexor activity was blocked thereafter for the duration of the photostimulation, thus preventing further steps and locomotion, but the locomotor rhythm was reset upon termination of photostimulation (Fig. 5a, b).

Sites evoking acceleration or delaying acceleration evoked an increase in the amplitude and frequency of both flexor and extensor bursts before SCI (Fig. 5c, d), which were lost after SCI. The ability of the descending glutamatergic reticulospinal drive to increase the burst amplitude of the flexor was lost for most stimulation sites after SCI, but was preserved in sites accelerating locomotion (Fig. 5e, g). Contrary to flexors, the ability after SCI to increase the recruitment of stance-promoting extensors was barely affected (Fig. 5f, g), which probably explains the higher number of sites stopping for the entire duration of photostimulation in the chronic phase of SCI (Fig. 4b). In summary, despite the loss of descending ipsilesional reticulospinal axons, the MRF continued to modulate locomotor rhythm and pattern after chronic SCI.

### MRF generates locomotion and/or steering after chronic SCI

**Fig. 6:**
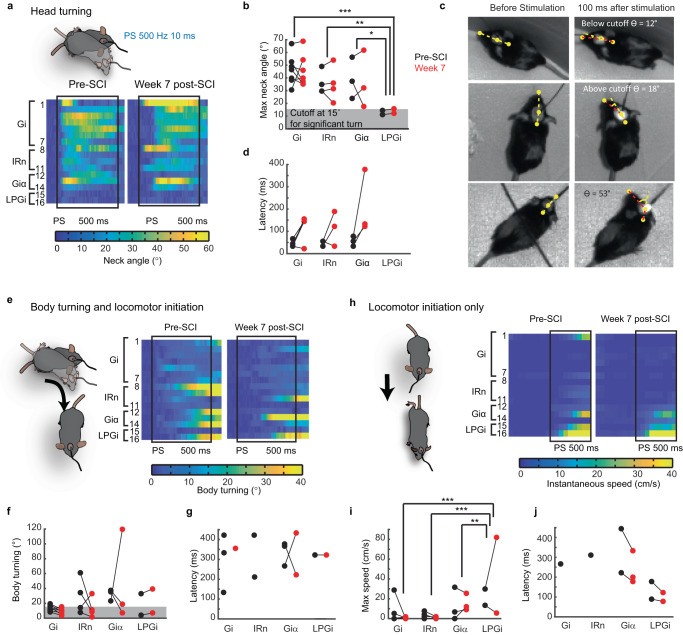
Effects of trains of photostimulation of the medullary reticular formation at rest in an open field. **a** Color-coded matrices of neck angular changes upon photostimulation (*n* = 16 mice, 10 trials per mouse). **b** Maximal neck angular changes during photostimulation. Gi, *n* = 7; IRn, *n* = 4; Giα, *n* = 3, LPGi, *n* = 2. Two-tailed two-way ANOVA: column effect, *F*_(3,22)_ = 9.168, *p* = 0.0004; row effect, *F*_(6,22)_ = 1.123, *p* = 0.38. Multiple comparisons tested with post hoc Tukey’s test. **c** Examples of neck turning showing the 15° cutoff for significant angular changes measured in (**d**), the latency of neck turning. Gi, *n* = 7; IRn, *n* = 4; Giα, *n* = 4; LPGi, none. Two-tailed two-way ANOVA: column effect, *F*_(2, 19)_ = 0.908, *p* = 0.420; row effect, *F*_(6,19)_ = 0.072, *p* = 0.998. **e** Color-coded matrix of body turning. **f** Maximal body angular changes. Gi, *n* = 7; IRn, *n* = 4; Giα, *n* = 3, LPGi, *n* = 2. Two-tailed two-way ANOVA: column effect, *F*_(3,22)_ = 1.900, *p* = 0.159; row effect, *F*_(6,22)_ = 0.780, *p* = 0.595. **g** Latency of body turns greater than 15°. Gi, *n* = 3 pre-SCI and *n* = 1 post-SCI; IRn, *n* = 2 pre-SCI and none post-SCI; Giα, *n* = 3 pre-SCI and *n* = 2 post-SCI; LPGi, *n* = 1 pre- and post-SCI. Two-tailed two-way ANOVA: column effect, *F*_(2,14)_ = 0.338, *p* = 0.719; row effect, *F*_(3,14)_ = 0.966, *p* = 0.436. **h** Color-coded matrix of speeds of locomotor bouts initiated by photostimulation. **i** Maximal speed of locomotor bouts. Gi, *n* = 7; IRn, *n* = 4; Giα, *n* = 3, LPGi, *n* = 2. Two-tailed two-way ANOVA: column effect, *F*_(3,22)_ = 10.87, *p* = 0.0001; row effect, *F*_(6,22)_ = 0.797, *p* = 0.582. **j** Latencies of locomotor bout initiation for speed >5 cm/s. Gi, *n* = 1 pre-SCI and none post-SCI; IRn, *n* = 1 pre-SCI and none post-SCI; Giα, *n* = 2 pre-SCI and *n* = 3 post-SCI; LPGi, *n* = 2 pre- and post-SCI. Two-tailed two-way ANOVA: column effect, *F*_(3,8)_ = 2.107, *p* = 0.178; column effect, *F*_(2,8)_ = 2.765, *p* = 0.122. Bin size is a video frame (11.1 ms) in (**a**) and (**e**) and averaged over 10 frames in (**h**). Gi gigantocellular nucleus, Giα gigantocellular reticular nucleus, alpha part, IRn intermediate reticular nucleus, LPGi lateral paragigantocellular reticular nucleus. Source data are provided as a Source Data file.

We next assessed whether photostimulation trains can still generate different types of behaviors after SCI in an open field. Before SCI, photostimulation trains of 500 ms triggered head turning in all mice except sites located in the LPGi (Fig. 6a, b and Table 1). Body turning was evoked toward the end of the photostimulation train in about half of mice from different sites (Fig. 6e, f). Initiation of moderate to high-speed locomotion was evoked mainly upon stimulation of the LPGi and GiV/α, whereas slow speed locomotion was sometimes generated by the IRn and rarely by the Gi (Fig. 6h, i and Table 1). Following SCI, maximal neck angle was obviously not affected (two-way ANOVA, effect of time, *p* = 0.369, Fig. 6b–d). Surprisingly, the amplitude and occurrence of body turning, which depends on the coordination of forelimbs, hindlimbs, and axial muscles, were also not affected (Fig. 6f, g, Chi-square test, *p* = 0.15). Locomotion was still generated by stimulation of the Giα and LPGi, but not by the Gi or the IRn (Fig. 6i). Latencies for head turning, body turning, and locomotor initiation were not significantly altered by the thoracic hemilesion. Overall, glutamatergic neuronal populations distributed through the MRF can generate various motor and locomotor functions before SCI^7,9–11,19^ that are preserved after SCI.

**Table 1:** Motor effect of trains of photostimulation

Motor behavior	Figure	Gi	IRn	GiA/V	LPGi
Neck turn	Fig. 6a–d	7/7 7/7	4/4 4/4	3/3 3/3	– –
Body turn	Fig. 6e–g	3/7 1/7	2/4 –	3/3 2/3	1/2 1/2
Locomotion initiation	Fig. 6h–j	1/7 –	2/4 –	2/3 3/3	2/2 2/2
Stops and deceleration	Fig. 4	6/7 7/7	3/4 1/4	– –	– –
Acceleration	Fig. 4	1/7 –	1/4 –	3/3 3/3	2/2 2/2

Number of mice expressing a given motor behavior on the total number of mice. Upper row, before spinal cord injury; lower row, 7 weeks after spinal cord injury. If there is no mouse expression a given behavior, an en dash is used for better visualization.

### MRF can improve stepping ability of chronically impaired SCI mice

**Fig. 7:**
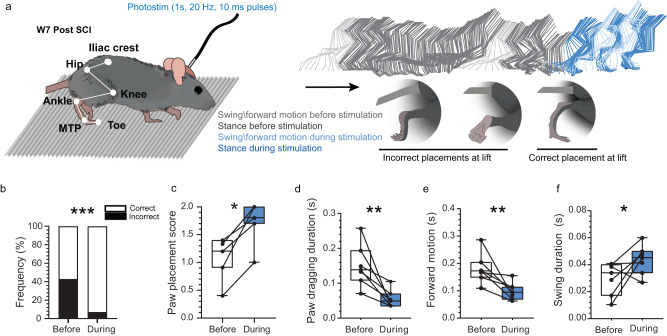
Locomotion can be improved by photostimulating the MRF. **a** Stick diagram of the ipsilesional hindlimb during treadmill locomotion illustrating chronic impairments during swing (light gray) and stance (dark gray), and improvement by photostimulation (blue). Below stick diagram, schematics of incorrect and correct paw placement. **b** Bar graphs to show effects of photostimulation on percentage of correct and incorrect paw placements (*n* = 7 mice, 3–5 trials per mouse). One-tailed Wilcoxon signed rank of correct placement, *W* = 28, *p* = 0.0078. **c** Paw placement score: 0 = dorsal contact, 1 = lateral contact, 2 = plantar contact. Individual points from the same mouse before and during photostimulation are connected. One-tailed Wilcoxon signed rank, *n* = 7 mice, *W* = 28, *p* = 0.0078. **d** Duration of paw dragging. One-tailed paired *t*-test, *n* = 7 mice, *t*_(6)_ = 3.730, *p* = 0.0049. **e** Duration of forward motion during incorrect placement. One-tailed paired *t*-test, *t*_(6)_ = 3.369, *p* = 0.0075. **f** Swing duration. One-tailed paired *t*-test, *t*_(6)_ = 2.051, *p* = 0.043. Statistical significance was tested with a Wilcoxon signed rank, *p value < 0.05, **p value < 0.01. Center of boxplots is the median, upper and lower limits of boxes are the first and third quartile respectively and the upper and lower limits of whiskers are the maxima and minima contained within the Tukey’s distance. Source data are provided as a Source Data file.

Despite some recovery (Fig. 1), some chronically impaired mice still performed misplacement on the dorsum or on the lateral side of their ipsilesional hindpaw up to 7 weeks after SCI (Fig. 7a). We tested a sub-group of mice (*n* = 7 mice) during episodes of treadmill locomotion at comfortable steady speed (7–25 cm/s, depending on the mouse). Using trains of photostimulation at 50 Hz for 1 s (Fig. 7a), it was possible to shift from dorsal to plantar contact (Supplementary Movie 1). Photostimulation improved the occurrence and the score of correct ipsilesional paw placements (Fig. 7b, c) by decreasing the duration of hindpaw dragging and forward motion (Fig. 7d, e) and increasing the swing phase duration with the toe off the ground (Fig. 7f). Taken together, these kinematic changes induced by MRF activation improved stepping ability of chronically impaired SCI mice.

### Priming the MRF can improve skilled locomotion after SCI

**Fig. 8:**
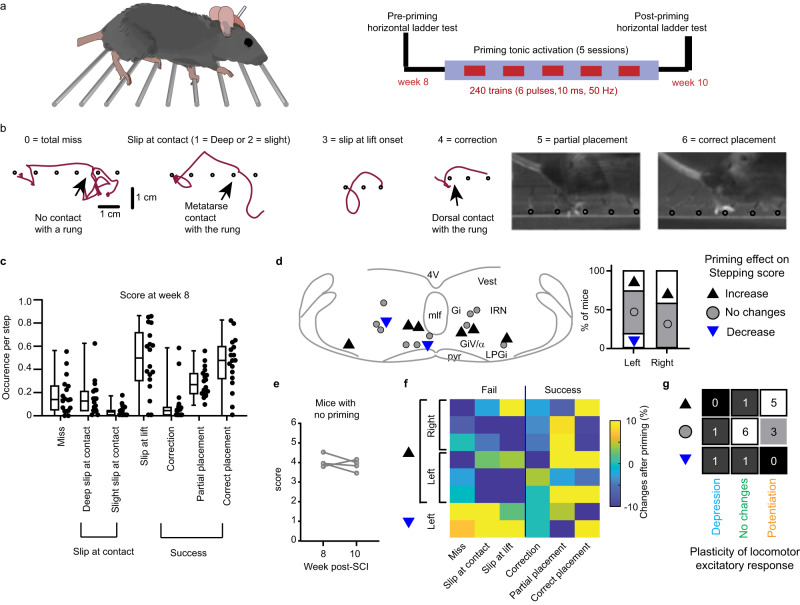
The MRF can be primed to improve skilled locomotion. **a** Experimental design for priming the descending drive to improve skilled locomotion. **b** Types of step during locomotion for scoring performance of the ipsilesional hindlimb on the horizontal ladder. **c** Boxplot of the percentage of steps for total miss, slip at contact (deep or slight), or lift and successful placement (correction, partial or correct) before priming (*n* = 18 mice). Data are averages from 18–35 steps over 3 passages. Center of boxplots is the median, upper and lower limits of boxes are the first and third quartile respectively and the upper and lower limits of whiskers are the maxima and minima contained within the Tukey’s distance. **d** Mapping of primed sites (one per mouse, *n* = 11 in the left MRF and *n* = 7 in the right MRF). Effect is coded by an arrowhead (downward in blue for decrease and upward in black for increase) and lack of effect by a gray circle. Percentages of effects are reported in a bar graph. The significance of the effect was determined with a two-tailed Mann–Whitney rank sum test of the score before and after priming. **e** Average score of mice with no priming and tested 2 weeks apart. Two-tailed Wilcoxon signed rank, *W* = −6, *p* = 0.375. Statistical significance was tested with a Wilcoxon signed rank. **f** Changes of occurrence of failures (misses, slip at contact and at lift) and successes (correction, partial and correct placement) for sites with significant changes. **g** Contingency of effects of priming vs. plasticity of excitation during locomotion. Depression in blue, no changes in green and orange in potentiation. Source data are provided as a Source Data file.

Given the correlation of the descending reticulospinal drive plasticity with spontaneous motor recovery after SCI (Figs. 2 and 3) and its preserved functional neuromodulation after chronic SCI (Figs. 4–7), we next evaluated whether priming tonic photoactivation of the MRF could boost functional outcomes after chronic SCI. Voluntary locomotion was evaluated on a horizontal ladder 8 weeks after SCI before and after a priming tonic photostimulation of glutamatergic neurons of the contra- or ipsilesional MRF for five sessions over 2 weeks in chronic SCI mice at rest (Fig. 8a). Before priming, the most common deficit observed was a foot-slip on rungs of the horizontal ladder at lift followed by partial placement of the paw on the wrong rung and misses. Correction and slips at contact, with (deep slip) or without (slight slip) loss of weight, were less frequent (Fig. 8c). Performance on the horizontal ladder was not influenced by the extent of the lesion as a whole (Supplementary Table 1). After five sessions of 20 min at the minimal intensity to evoke muscle twitching at rest over a period of 2 weeks, locomotion was reassessed on a horizontal ladder. Priming tonic photostimulation on both sides could improve the global skilled locomotor score (*n* = 3/7 mice for the right side and *n* = 3/11 mice for the left MRF, two-tailed Fisher’s exact test, *p* = 0.63). Positive effects were observed for the Gi and the LPGi, and detrimental effects for the left IRn and Giα/GiV (Fig. 8d). No changes were observed without priming (Fig. 8e). In general, increased score was due to slips at contact being replaced in favor of more partial and correct paw placements. In opposition, decreased score was related to more misses and foot slips (Fig. 8f). Almost all mice (*n* = 5/6) showing an improvement were associated with potentiation of their locomotor excitatory responses, whereas those without improvement were associated either with depression or no change (two-tailed Fisher’s exact test, *p* = 0.043, Fig. 8g). These results highlight the potential of priming tonic activation at rest of glutamatergic MRF neurons to improve functional outcomes after chronic SCI.

## Discussion

Combining electromyographic and kinematic analysis with optogenetic manipulation in freely behaving mice, we have investigated the functional plasticity of the descending glutamatergic reticulospinal drive of the MRF to spontaneous locomotor recovery after SCI and also assessed whether its stimulation could boost locomotor recovery after chronic SCI. Understanding the organization, function, and plasticity of glutamatergic neurons of the MRF and its reticulospinal drive to the locomotor circuit after SCI is critical to enhancing rehabilitation in patients with chronic SCI. Our results reveal that functional plasticity of the glutamatergic reticulospinal drive is independent of the stimulation site throughout the MRF, but is largely determined by its initial strength on the lumbar locomotor circuit before SCI. Photostimulation trains of the most dorsal sites of the MRF (Gi and IRn) recruit flexor muscles after SCI less efficiently, thus leading to a higher occurrence of locomotor arrests, whereas stimulation of the most ventral sites (GiV/α and LPGi) increases a rhythmic motor drive in flexor and extensor muscles of the ipsilesional hindlimb, thus promoting locomotion after SCI. In addition to spontaneous plasticity, photostimulation trains also improved correct paw placement of the ipsilesional hindlimb of chronically impaired SCI mice during locomotion. Finally, as a therapeutical approach, priming tonic photostimulation of chronic SCI mice at rest for 2 weeks improved skilled locomotion on a horizontal ladder. In summary, stimulation protocols of discrete MRF nuclei could improve motor recovery in SCI patients.

Pharmacogenetic inhibition of glutamatergic neurons of the ventral part of the Gi has been reported to abolish spontaneous locomotor recovery after a severe thoracic contusion^5^. Similarly, pharmacogenetic silencing of unidentified reticulospinal Gi neurons projecting either above or below the lesion also impairs spontaneous motor recovery from a lateral cervical hemisection^15^, thus suggesting that stimulation of direct and/or indirect (i.e., ipsi and/or contralesional) reticulospinal pathways could improve locomotor recovery after SCI.

Incomplete spinal cord injury activates various mechanisms of plasticity within spinal networks^1,23,24^, which could be recruited by spared supraspinal pathways. Given their excitatory reticulospinal drive on motor control and locomotion^7^, we focused in the current study on glutamatergic neurons of the MRF. As previously shown in intact settings^7,20^ and more recently in SCI mice^25^, using a paradigm of 10 ms photostimulation pulses allowed us to probe changes in reticulospinal efficacy over time at rest and during locomotion after SCI.

Although unexpected, changes in reticulospinal efficacy did not depend on the stimulation site throughout the MRF. However, acute and chronic changes depended on the initial strength of the reticulospinal drive prior to SCI: The stronger the motor responses before SCI, the stronger they were depressed after SCI; the weaker the motor responses prior to SCI, the stronger they were potentiated after SCI. Given their importance and necessity after SCI^5,15^, our photostimulation studies show that the descending drive of glutamatergic neurons of the MRF is preserved, albeit with some changes, and could presumably contribute to spontaneous motor recovery after SCI.

Given that neuroanatomical changes are unlikely to occur within a week, the most likely scenario is that spared reticulospinal synapses might have undergone homeostatic plasticity^26,27^ by enhancing their descending drive during the acute SCI phase. Because of a reciprocal connectivity between reticulospinal neurons and reticular interneurons within the medullary reticular formation^28^, synaptic and intrinsic plasticity could also occur at the supraspinal level. In addition to synaptic plasticity of reticulospinal pathways during the chronic phase, potentiation or recovery of motor responses likely also depend on neuroanatomical plasticity^13,14,29^ as well as on a higher intrinsic excitability of the spinal locomotor network^30^.

Using photostimulation trains, our results highlight the important contribution of glutamatergic neurons in the vicinity of the Gi to deceleration and stop, and that of the LPGi and GiV/α to locomotor initiation and acceleration after SCI. As previously shown in intact animals^19^ and from in vitro brainstem spinal cord preparations isolated from neonatal mice^16^, photostimulation trains delivered above glutamatergic neurons of the LPGi generated locomotion with an increased rhythmic drive in hindlimb flexor and extensor muscles before and after chronic SCI.

Except for discrete sites, photostimulation trains delivered above glutamatergic neurons within the Gi induced overall transient deceleration or locomotor arrest prior to injury, but they evoked systematic locomotor stop after chronic SCI, thus suggesting potentiation of reticulospinal pathways promoting stops. Although glutamatergic V2a Gi neurons have been called stop cells for their capacity to stop locomotion by inhibiting premotor activities^9^, in contrast our results show that stimulation of all glutamatergic neurons within the Gi and the IRn stopped locomotion by an initial co-activation in flexor and extensor motor pools followed by a sustained extensor activity. Although virtually all V2a populations of the MRF are glutamatergic^8,9^, it is still unknown whether all glutamatergic Gi neurons of the MRF are V2a, thus suggesting the existence of a non-V2a glutamatergic Gi population that could disfacilitate flexor motor activity and maintain extensor motor activity during locomotor stop. Furthermore, this pathway appears to be potentiated in chronic SCI mice.

Interestingly, stimulation of some discrete sites located within the Gi area also evoked delayed acceleration or locomotor initiation prior to SCI, in line with recent in vitro neonatal brainstem spinal cord preparations^16^. However, this pro-locomotor effect driven by glutamatergic Gi neurons was lost after SCI. At the interplay between the most ventrolateral and dorsomedial regions of the MRF, stimulation of glutamatergic neurons in the vicinity of the Giα/V generated a transient deceleration followed by an acceleration before and after chronic SCI. These results suggest the existence of an intermediate reticulospinal population between the Gi and LPGi that could generate deceleration or acceleration phases during transition of gaits.

In summary, although the LPGi and Giα/V maintained their pro-locomotor effects after chronic SCI, Gi generating transient deceleration (with or without delayed acceleration) prior to injury shifted only to locomotor stop effects after chronic SCI, thus supporting functional plasticity.

One of our most intriguing results was the lack of functional lateralization upon photostimulation pulses or trains. In line with bilateral motor responses evoked by glutamatergic Gi neurons in intact mice^7^, photostimulation pulses evoked motor changes in the ipsilesional hindlimb in resting and walking mice before and over time after SCI, regardless of their contra- or ipsilesional stimulation sites. In contrast, the capacity to modulate locomotion was rather dependent on the ventro-dorsal location, with the most ventral sites (LPGi and GiV/α) evoking locomotion and the most dorsal sites (Gi and IRn) stopping it. Interestingly, despite an extensive ipsilateral lesion, ipsilesional motor responses were evoked with a 2 ms delay 1 week after SCI, thus suggesting that the cervical propriospinal or contralesional MRF network might relay ipsilesional reticulospinal inputs. Our results showing that glutamatergic neurons of contra- and ipsilesional MRF nuclei maintain their access to the lumbar locomotor circuit below the lesion site were therefore unexpected and surprising, suggesting a high degree of divergence in their axonal paths but also of convergence with respect to the spinal locomotor circuit.

Anatomically, an anterograde tracing study has shown that reticulospinal pathways branch extensively and exhibit a very high density of synaptic contacts, even stronger than corticospinal ones, onto propriospinal interneurons in intact settings^31^, thus suggesting that the reticulo-propriospinal component above the lesion could be the site of an early and ongoing homeostatic plasticity but also long-term anatomical plasticity. Retrograde tracing studies have also shown that a substantial proportion of Gi neurons send multiple axonal collaterals to both cervical and lumbar enlargements in control rodents^32,33^. After SCI, reticulospinal neurons increase their axonal collaterals across the midline to innervate the ipsilesional spinal gray matter below the lesion^13,34^, and they can also form new synaptic contacts onto propriospinal interneurons above and below the lesion^12,14^. Finally, pharmacogenetic inhibition of either contralesional reticulospinal neurons of the Gi projecting below the lesion or ipsilesional neurons projecting above the lesion impair spontaneous motor recovery after SCI^15^. In light of the aforementioned studies, our findings herein suggest that ipsilesional glutamatergic reticulospinal pathways, even injured, could access the lumbar locomotor circuit presumably through the recruitment of cervical descending propriospinal pathways bypassing the lesion. In support of this hypothesis, pharmacogenetic inhibition of cervical propriospinal pathways impairs spontaneous motor recovery after SCI^35^. Although of lower density^11^, contralateral glutamatergic reticulospinal axons could also access the spinal locomotor circuit below the lesion by crossing the midline^13^ or simply by recruiting local spinal commissural interneuronal pathways. Further studies will be necessary to investigate neurophysiological and neuroanatomical changes of these direct and indirect reticulospinal pathways after SCI.

Neuromodulation of glutamatergic neurons of the MRF can improve stepping after chronic SCI and its priming at rest can strengthen the descending reticulospinal drive, which in turn improves skilled locomotion. Neuromodulatory approaches have been investigated over the last decade to improve functional outcome after SCI^1^. Epidural electrical stimulation of the lumbar spinal cord below the lesion can improve functional locomotor outcome in animal models, but also in individuals with SCI^4,36–38^. Neural mechanisms underlying epidural electrical stimulation of the spinal cord involve the recruitment of glutamatergic *Vsx2/Hox10* spinal interneurons (V2a), which also receive reticulospinal inputs from the ventral MRF^39^, which receives cortical and midbrain inputs^5,19^. Recently, glutamatergic neurons of the cuneiform nucleus have been reported to be more efficient than those of the pedunculopontine nucleus in promoting spontaneous motor recovery from SCI, and their stimulation enhances functional outcome after chronic SCI^25^, thus suggesting that glutamatergic neurons of the LPGi and GiV/α could relay glutamatergic cuneiform inputs important to motor recovery after SCI.

In conclusion, our results show that the plasticity of the glutamatergic reticulospinal drive occurs alongside spontaneous motor recovery from SCI and maintains its function in modulating locomotor pattern and rhythm (i.e., initiation, acceleration, deceleration, and stop). Finally, its priming stimulation at rest can induce outlasting functional outcomes during skilled locomotion after chronic SCI. Given its deep location within the brain, the MRF is still a difficult structure to access. However, emerging technologies such as upconversion nanoparticle-mediated optogenetic stimulation at near-infrared wavelengths^40^ could offer interesting non-invasive ways to probe, neuromodulate, and condition neural activity of the MRF to promote functional outcomes in individuals with SCI.

## Methods

All experiments were approved by the committee for animal care of Université Laval (Comité de protection des animaux de l’Université Laval, protocol number 19-027) and followed guidelines of the Canadian Council on Animal Care.

### Animal model

C57/BL6J VGluT2-cre (The Jackson Laboratory, strain 028863) female mice (*n* = 26) were used. A subset (*n* = 8) of VGluT2-cre mice crossed with Ai32 mice (The Jackson Laboratory, strain 028863). Mice were 2–3 months old at the beginning of experimentation. After surgery, mice were housed alone or in groups of 2–3 individuals. They were kept on a 12 h dark 12 h light cycle, had ad libitum access to food and water and were supplied with nesting and bedding materials (Neslets, Aspen shaving and Enviro-Dri). Temperature was kept at 23 ± 2 °C and humidity at 50 ± 5%. Air was changed 8–12 times per hour.

### Virus

VGluT2-cre mice were injected either with an AAV9-Ef1a-DIO-hChR2(H134R)-YFP (Addgene plasmid #35507, *n* = 22 mice) or AAV9-EF1a-DIO-hChR2(H134R)-mCherry (Addgene plasmid #20297, *n* = 4 mice). Viruses were suspended in phosphate buffer saline (320 nM NaCl) with 5% Sorbitol 5% and 0.001% pluronic F-68. Titer was 9x10E12 gc/ml. Both viruses were developed with the molecular tools platform at the Canadian Neurophotonics Platform (Quebec City, Canada).

### Surgical procedures

Surgeries were performed under isoflurane anesthesia (1–3%). All incision and pressure points were injected subcutaneously with lidocaine-bupivacaine. Mice were placed in a stereotaxic frame. A craniotomy of 2–3 mm in diameter was performed to expose the brain prior to injection. Targeted coordinates ranged from anteroposterior bregma −6.0 to −7.3 mm, mediolateral 0.1 to 1.6 mm, and depth from 5 to 6.3 mm (the distance from the ventral surface is reported in the main text). A volume of 100 nl of virus was injected at a rate of 50 nl/min with a glass pipette attached to a Nanoliter 2020 Injector (World Precision Instruments). Pipette was left in place for about 2 min before withdrawal. A self-assembled optical implant built with multimodal optical fiber (100 µm for VgluT2-cre::Ai32 and 200 µm for VGluT2-cre mice with AAV9 injections) was lowered 0.1 mm above the injection site. Implantation was performed either on the left (*n* = 15) or the right side (*n* = 19). The optical implant was secured on the head with screws in the skull and with dental acrylic. Mice were injected with sustained-release buprenorphine for long lasting analgesia.

Three weeks after surgery, mice were tested on the treadmill. The efficacy of implanted sites was evaluated with kinematic and EMG recordings. After 1 month of rest since the first surgery, mice were anesthetized once more with isoflurane. The spinal cord at thoracic levels T7-T11 was exposed by laminectomy. A lateral hemisection was performed on the left side for all mice with a gauge 30 needle. A 1 mm piece of Surgicel was inserted into the transected tissue. Back muscles and skin were sutured back. Mice were returned to their cage and paresis of the left hindlimb was observed upon waking from anesthesia. Bladder was manually emptied twice daily for a period of 2–4 weeks.

### Kinematic and EMG recordings

All mice were trained to walk on a 30 cm treadmill (Exer 3/6 Treadmill, Columbus Instruments or LE8700, Panlab/Harvard Apparatus) at a walking speed of 15 to 25 cm/s before SCI. This moderate speed range was used for comparison purposes with subacute locomotor capabilities. Testing began when the mouse walked most of the time at a steady pace in the front third of the treadmill lane. Mice were filmed bilaterally with Genie GigE cameras (Teledyne Dalsa) and recorded with Streampix (NorPix) for offline analysis.

For treadmill kinematic analysis, mice were briefly anesthetized with 1% isoflurane to label the iliac crest, hip, ankle, metatarsophalangeal (MTP) joint, and the toe with an oil-based paint marker (Sharpie). Kinematic recording was obtained at a frequency of 250 frames s^−1^ on both sides. Percutaneous EMG recordings of the Tibialis anterior and Gastrocnemius lateralis on both sides were acquired with insulated nickel-chromium duplexed wires (50 μm diameter, California Fine Wire, CFW2027239) inserted with 30 G needles. EMG signals were amplified with Model 3600 extracellular amplifier (A-M Systems) and recorded on Spike2 (Cambridge Electronic Design).

A subset of mice (*n* = 16) was tested for 1-s trains of photostimulation (1 s at 50 Hz, 10 ms pulse duration) during treadmill locomotion. The same mice were tested in an open-field arena of 60 cm in diameter. The patch-cord was connected to a rotary joint. For assessment of locomotor initiation, each mouse was placed at the center of the open field and was filmed from above at 90 frames s^−1^. A 500 ms (50 Hz, 10 ms spike duration) train of photostimulation was delivered when the mouse was at rest for at least 1 s.

A subset of mice (*n* = 18) was tested for skilled locomotion on the horizontal ladder. The horizontal ladder was made of evenly space rungs (1 cm). The ladder was 90 cm in length but mice were scored while crossing a 30 cm section at the center of the ladder. Camera was positioned on one side and the left and right sides were filmed separately as the mouse walked back and forth on the ladder (3 passes per side).

### Optogenetic stimulation and priming tonic activation

To activate MRF sites, the optical implant was connected to a 473 nm solid-state laser diode (Laserglow Technologies). The threshold was sought at rest by 5% increments of the laser current drive (maximal power 63 mW at 100%). The threshold ranged from 55% (irradiance of 50 mW mm^−2^ for 200 µm optical implant, 40 mW mm^−2^ for 100 µm optical implant) to 80% (irradiance of 700 mW mm^−2^ for 200 µm optical implant, 450 mW mm^−2^ for 100 µm optical implant). During locomotion, this threshold was increased by 5% to compensate for gating of the descending drive^7^. Data reported in this study were obtained at constant laser intensity for the different timepoints.

The underlying concept of our priming protocol was to promote context-independent long-term plasticity. To condition the descending pathway after SCI, each mouse received trains of low-intensity stimulation while quietly resting. The intensity of stimulation was evaluated as the lowest burst (3–6 pulses, 10 ms duration, 20–50 Hz) activating EMG response that did not change the animal’s apparent awareness and thus likely did not cause any discomfort. Bursts were delivered every 5 s for 20 min while the animals were quietly at rest inside their cages. Each mouse received five sessions of priming over 2 weeks. Two days after the last day of priming, mice were tested for the second time on the horizontal latter to assess the impact of priming.

### Histology

Mice were deeply anesthetized with ketamine-xylazine (100 mg kg^−1^ and 10 mg kg^−1^) or isoflurane 4% and transcardially perfused with 4% paraformaldehyde in phosphate buffer 0.1 M NaCl 0.9% (PBS). Brains and spinal cord tissues were harvested and kept in sucrose 30% in PBS solution. Tissues were frozen on dry ice in OCT compound (Tissue-Tek) and sectioned at 40 µm with a cryostat (Leica). Images were taken on an Axio Imager M2 microscope using ZEN2 software (Zeiss).

### Histological analysis

The section with the maximal depth of the optical fiber was identified and imaged in ZEN2. To determine the position of optical implants, the section with the deepest track was imaged for offline measurements of the distance of the center of the tip of the implant from the brainstem midline and from the base of the pyramidal tract. Anteroposterior position was determined using a mouse brain atlas^41^. Of the 22 mice that were injected with an AAV-ChR2-eYFP, the signal was found in 20 of them. The signal was very faint for mice injected with AAV-ChR2-mcherry and was therefore not analyzed (*n* = 4). The eYFP signal was variable from mouse to mouse, but focusing on the eYFP signal we observed a small photobleached region of about 100–200 microns just below the tip. To quantify this (Supplementary Fig. 2), we extracted the x-y coordinates of each ChR2-eYFP positive cell and the position of the tip of the optical implant using ImageJ. The distance from these cells to the tip of the optical implant was computed in Matlab for each mouse. The distribution of neurons around the tip for all mice was pooled and shows that this photobleached region of about 100–200 µm matched previous report of bleaching of ChR2-eYFP^42^ and GFP signals^43^.

The section with maximal extent of lesion was identified and imaged in ZEN2. Data were imported in ImageJ to measure the area of the spared white matter on the left (ipsilesional) and right (contralesional) side. If the left side was not completely lesioned, we computed the ratio of spared left area on the right area, converted it to percentage and subtracted it from 100% to obtain the lesion extent. When lesion encompassed the right side, we computed the ratio of damaged area on the size of the funiculus (intact + damaged) and converted it to percentage. The boundary between ventral and lateral funiculi was determined as an oblique line (45 degrees in relation to the dorsoventral axis) originating from the lower and more lateral part of the ventral horn and running outward.

### EMG analysis

EMG signals were first processed in Spike2. After high-pass filtering (FIR filter, 270 Hz), motor spikes were detected using WaveMark as negative deflections crossing a threshold (5 times the background noise level, usually about 15–25 µV) in a −0.2 to 0.3 ms sliding time windows. Motor units and high-pass filtered EMGs were exported to Matlab.

For responses at rest (Fig. 2), the number of spikes evoked by photostimulation was computed for each trial. Averaged baseline activity and its standard deviation were evaluated 0.5 s before photostimulation to determine the threshold with bins of 10 ms. Onset threshold was defined as the averaged baseline plus twice the standard deviation, while termination was defined as the average baseline plus the half amplitude of the response. Crossing of the threshold for less than 20 ms was considered a false crossing. Latency was calculated in spike2 on an averaged peristimulus time histogram with bins of 3 ms. The number of motor spikes was evaluated in the half-amplitude time window.

For response during locomotion (Fig. 3), step cycles were identified with the LTA bursting, whose onset was automatically detected using a z-transformed trace and the density of motor spikes in a 1-s time window. The phase of photostimulation within the step cycle was computed. If no burst was detected a second before photostimulation, the trial was tagged as occurring during a stop or a paw drag (based on kinematic data). The number of motor spikes in a 50 ms time window before and after onset of photostimulation was computed. The response was defined as the difference between after (a mixture of background and response) and before (purely background activity). We referred to this response as motor spike density (spikes per 50 ms).

To determine the direction of plasticity, pre-SCI responses were used as a baseline to compare the responses at week 1 and week 7 using Mann–Whitney rank sum test (distributions were not normal as assessed with a Kolmogorov–Smirnoff test). In absence of statistical changes, responses were deemed unchanged, depressed if smaller, or potentiated if larger.

### Kinematic analysis

Locomotor abilities on the treadmill was evaluated using a non-linear score^44,45^.

Detection of the markers on iliac crest, hip, ankle, MTP, and toes was carried out using DeepLabCut. For this purpose, 500 random frames were selected out of videos and used to create the model of analysis using mobileNet backbone. Initiation of locomotion was assessed in the open field and analyzed using DeepLabCut. To create the model based on the ResNet 101 backbone model, 850 frames were selected. The model was trained to detect the position of the tail base to monitor the walking speed and position of the snout, neck, and middle of the body axis to assess head turning. The output of DeepLabCut was imported and corrected in custom-designed software (graciously provided by Drs. S. Rossignol and T. Drew at Université de Montréal). Measurements and final visualizations were completed in Matlab.

To assess skilled locomotion, we used a scoring system for the horizontal ladder^46^. In this method steps were scored according to the correctness of lifts and contacts: total miss (0), slip at contact (deep = 1, slight = 2), slip at lift (3), and correct placements (with correction = 4, partial placement = 5 and correct placement = 6). The scores received by each hind limb during 3 passages were averaged and reported as the average score. Additionally, we reported the percentage of occurrence for each type of foot fault as the number of times each fault was observed divided by the number of steps. Each step was counted as the interval between two lifts.

Paw placement correction was assessed by measuring the duration of forward motion of the paw from the time point when the angle between ankle, MTP, and toes passed 90° to the next contact. Swing was considered as part of forward motion during which toes were not in contact with the ground. The difference between the durations of forward motion and swing was considered as dragging.

### Statistics

Because responses in VgluT2-cre::Ai32 mice were not different from the virally injected VgluT2-cre mice, data were pooled. All statistical testing was performed with functions in Matlab. Specific tests are addressed in figure legends. Normality was tested with Kolmogorov–Smirnov test to choose parametric (two-way ANOVA) vs. non parametric tests (Wilcoxon signed rank, Mann–Whitney, Friedman). All tests were two-sided. *F*-tests were run on independent and dependent variables for linear regression. When significant, slope and intercept were estimated with the fitlm function. Multiple linear regressions (Supplementary Table 1) were computed with Prism 10 (GraphPad).

### Reporting summary

Further information on research design is available in the Nature Portfolio Reporting Summary linked to this article.

### Supplementary information

Supplementary information

Peer Review File

Reporting Summary

Supplementary Video 1

### Source data

Source Data

## Data Availability

EMG data and coordinates of AAV recombination generated in this study have been deposited in the collection “Data for MRF SCI manuscript” on Figshare^47^ (10.6084/m9.figshare.c.6925099.v1). Source data are provided with this paper.

## Code availability

Matlab scripts for EMG analysis are available as the package EMGanalysis_MotorSpikes (v1.0.2) (10.5281/zenodo.8206041).
