# Supplementary material for: Functional plasticity of glutamatergic neurons of medullary reticular nuclei after spinal cord injury in mice
Source: Nat Commun. 2024 Feb 20;15:1542. doi: 10.1038/s41467-024-45300-4 (PMC10879492; doi:10.1038/s41467-024-45300-4)
Supplement: Supplementary file 1 — Supplementary information [file 41467_2024_45300_MOESM1_ESM.pdf]

Functional plasticity of glutamatergic neurons of medullary reticular nuclei after spinal cord injury in mice

Supplementary information

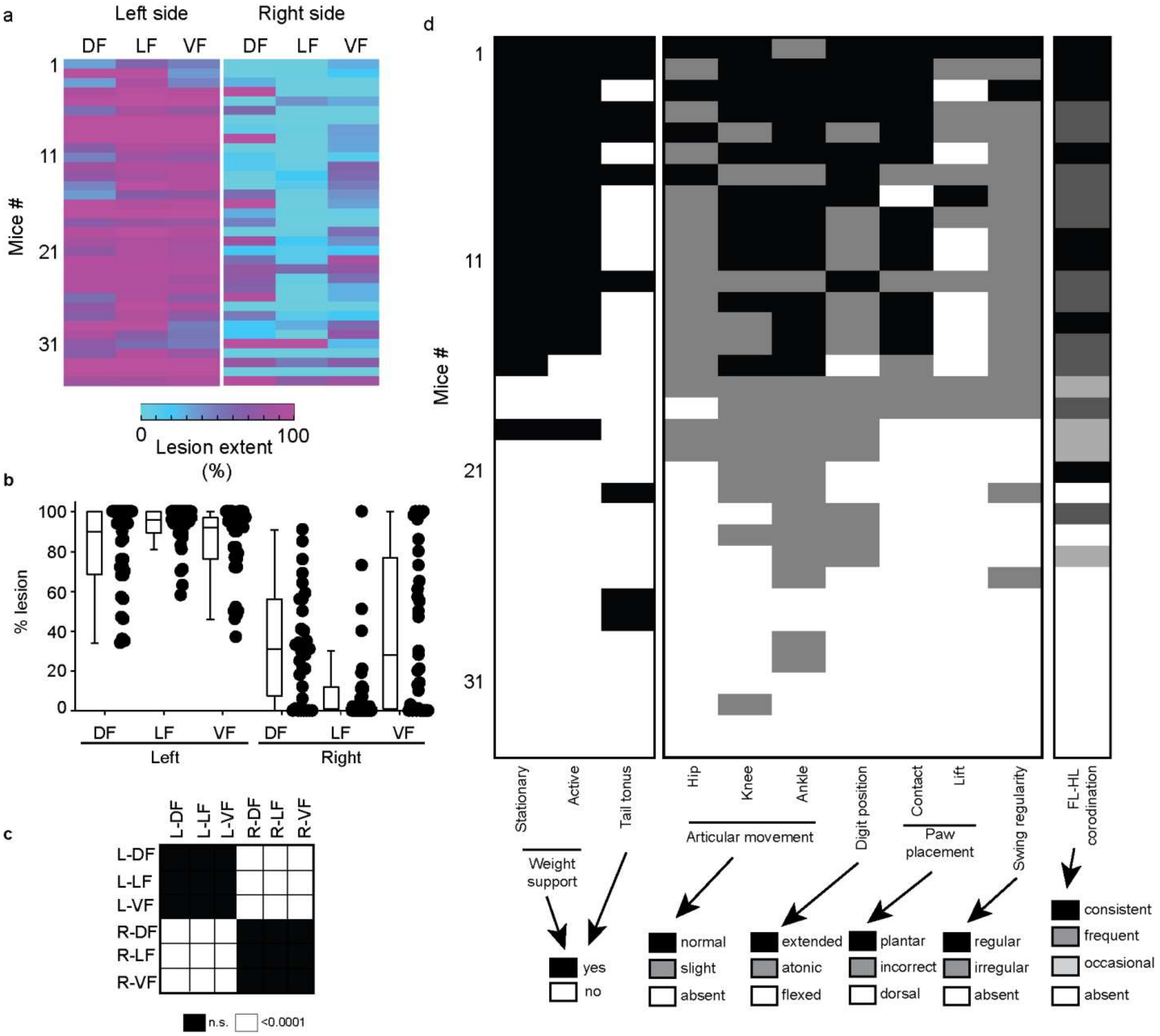

Supplementary Figure 1. Lesion extent does not correlate with stereotyped locomotor abilities

a, color-coded matrices of white matter lesion extent for each mouse (n = 34 mice) DF, dorsal funiculus; LF, lateral funiculus; VF, ventral funiculus; LH, left hindlimb; RH, right hindlimb. **b**, boxplot of the extent of lesioned white matter on the right and left side. Bartlett's test, p<0.0001, Two-tailed Kruskal-Wallis test, $\chi^2 = 107.6$, p < 0.0001. **c**, results of post-hoc Tukey test presented in a grayscale matrix. **d**, grayscale matrix of the detailed locomotor scoring. Source data are provided as a Source Data file.

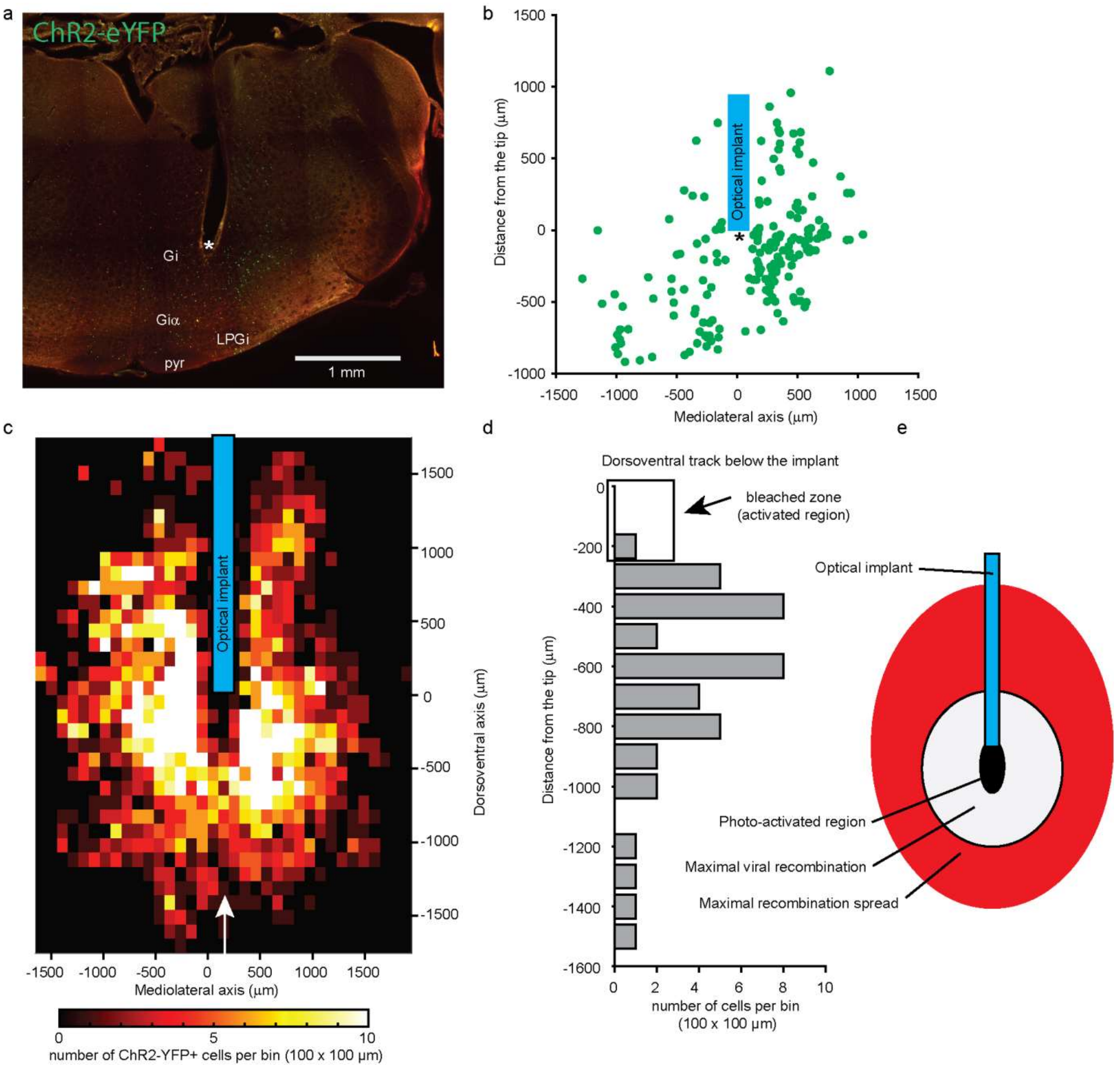

Supplementary Figure 2. Extent of the viral ChR2-eYFP recombination

a, low-magnification image of a brainstem section illustrating the track of the optical implant in the Gi nucleus. Cells expressing the ChR2-eYFP are in green against a red autofluorescent background (no signal). *, tip of the optical implant; Gi, gigantocellular reticular nucleus; Giα, gigantocellular reticular nucleus, alpha part; LPGi, lateral paragigantocellular reticular nucleus; pyr, pyramidal tract. **b**, position of cells from the tip of the optical implant shown in a. **c**, color-coded matrix of ChR2-eYFP+ cell density in spatial bins of 100 μm^2 with the tip of the optical implant as the origin (n = 20 mice). White arrow indicates a vertical line below the optical implant referred in d. **d**, bar graph of the number of cells in a vertical line below the implant to evidence a bleached region spanning 100-200 μm^2, which we define as the photoactivated region. **e**, schematic representation of the implant, the presumed photo-activated region, the maximal viral recombination zone, and the maximal viral spread. Source data are provided on

figshare as the dataset “AAV ChR2-eYFP recombination coordinates” [https://doi.org/10.6084/m9.figshare.c.6925099.v1].

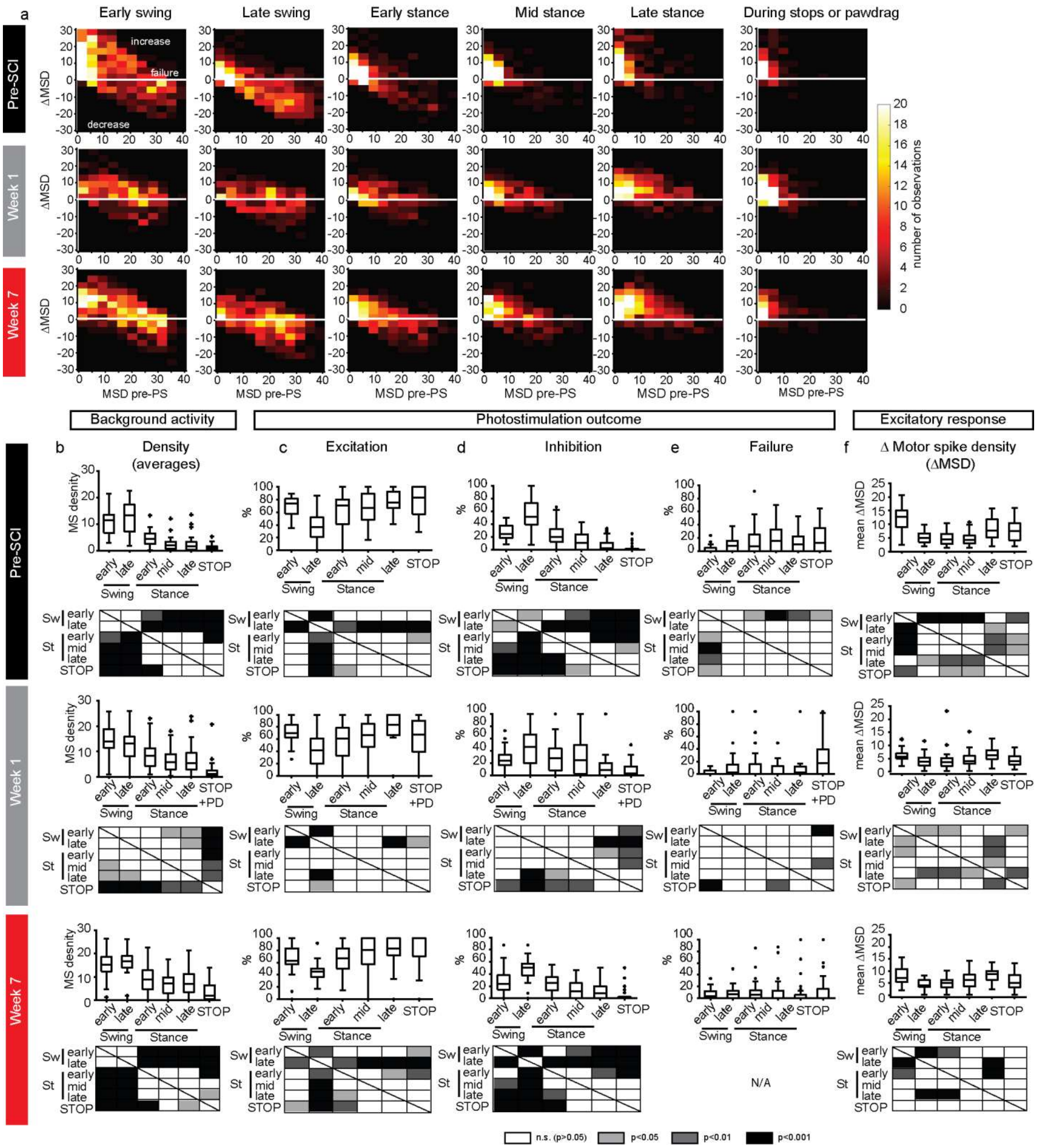

Supplementary Figure 3. LTA background activity and types of photostimulation outcome during locomotion before, one week after, and seven weeks after SCI

a, color-coded matrices of responses (ΔMSD) vs. background activity (MSD Pre-PS) during all phases and during stops (+ paw drag at week 1). Data from all mice (n = 34) were pooled. **b**,

boxplots of motor spike density (MSD, number of motor spikes in the 50 ms time window pre-photostimulation). Effect of phase pre-SCI, two tailed Kruskal-Wallis, $\chi^2 = 122.9$, $p < 0.0001$; week 1, two tailed Kruskal-Wallis, $\chi^2 = 68.14$, $p < 0.0001$; week 7, two-tailed one-way ANOVA, $F_{(5,29)} = 29.5$, $p < 0.0001$. **c**, boxplots of percentages of excitatory responses. Effect of phase pre-SCI, two tailed Kruskal-Wallis, $\chi^2 = 56.19$, $p < 0.0001$; week 1, two-tailed one-way ANOVA, $F_{(5,29)} = 5.49$, $p < 0.0001$; week 7, two-tailed one-way ANOVA, $F_{(5,29)} = 13.5$, $p < 0.0001$. **d**, boxplots of percentages of inhibitory responses. Effect of phase pre-SCI, two tailed Kruskal-Wallis, $\chi^2 = 118.1$, $p < 0.0001$; week 1, two tailed Kruskal-Wallis, $\chi^2 = 40.5$, $p < 0.0001$; week 7, two-tailed one-way ANOVA, $F_{(5,29)} = 30$, $p < 0.0001$. **e**, boxplots of percentage of failures of photostimulation to induce changes in MSD. Effect of phase pre-SCI, two tailed Kruskal-Wallis, $\chi^2 = 20.6$, $p = 0.001$; week 1, two tailed Kruskal-Wallis, $\chi^2 = 21.0$, $p = 0.0008$; week 7, two tailed Kruskal-Wallis, $\chi^2 = 6.6$, $p = 0.252$. **f**, motor spike density response (ΔMSD). Effect of phase pre-SCI, two tailed Kruskal-Wallis, $\chi^2 = 122.9$, $p < 0.0001$; week 1, two tailed Kruskal-Wallis, $\chi^2 = 68.1$, $p < 0.0001$; week 7, two-tailed one-way ANOVA, $F_{(5,29)} = 29.5$, $p < 0.0001$. Below each boxplot are the results of post-hoc Tukey's HSD tests (grayscale for p-values). Source data are provided as a Source Data file and on figshare as the dataset "Data and script for EMG response during locomotion (10 ms)" [https://doi.org/10.6084/m9.figshare.c.6925099.v1].

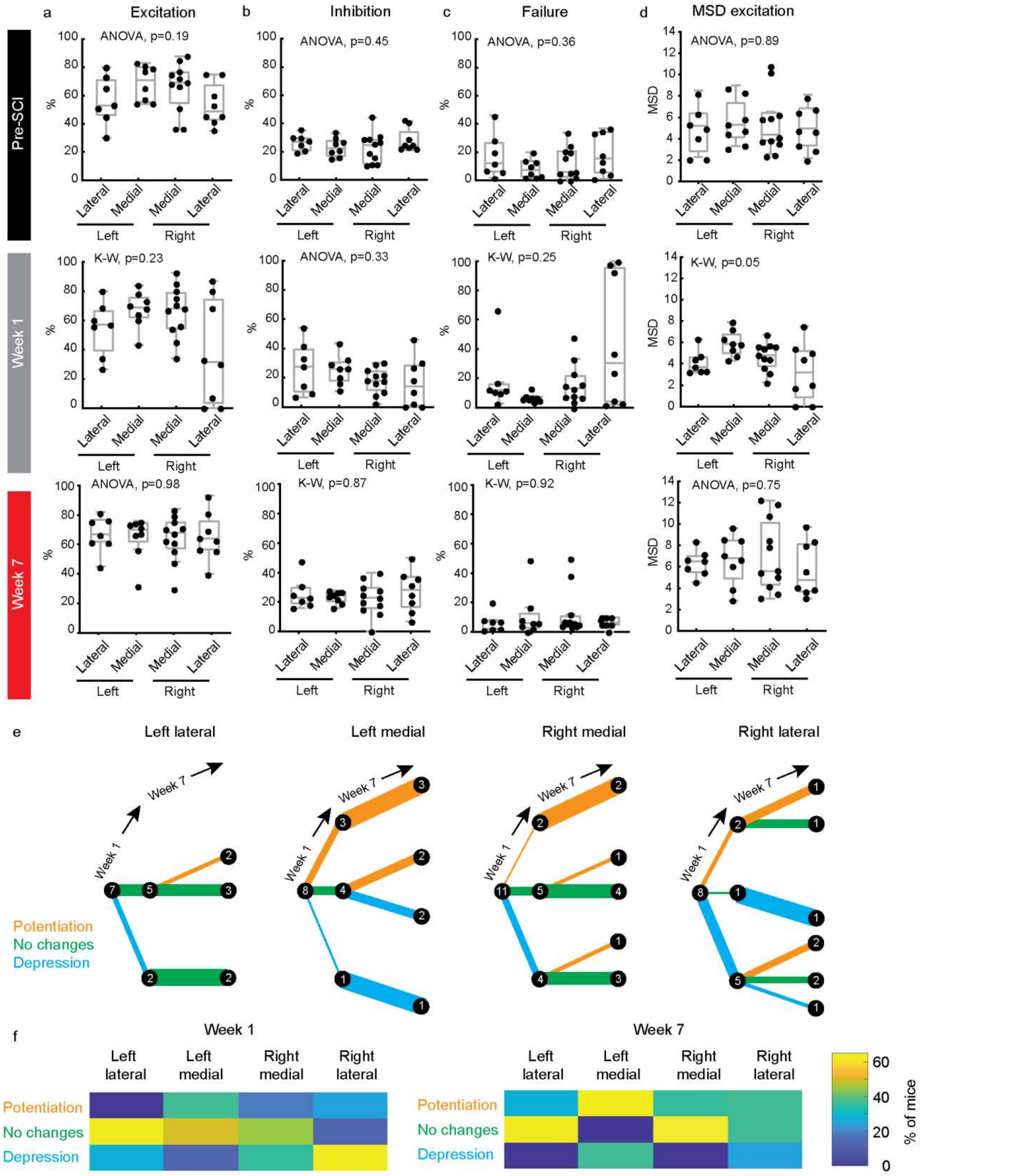

Supplementary Figure 4. Distribution of excitation, inhibition, failure, motor spike density (MSD) responses, and plasticity across medullary reticular groups

a, boxplots before (top), one week (middle) and seven weeks after (bottom) SCI of the percentage of trials showing an excitatory response across mediolateral groups. Lateral left side, n = 7 mice; medial left side, n = 8 mice; medial right side, n = 11 mice; lateral right side, n

= 8 mice. Effect of phase pre-SCI, two-tailed one-way ANOVA, $F_{(3,30)}$ = 1.69, p = 0.191; week 1, two-tailed Kruskal-Wallis, χ^2 = 4.34, p = 0.227; week 7, two-tailed one-way ANOVA, $F_{(3,30)}$ = 0.05, p = 0.984. **b**, boxplots of percentage of trials showing an inhibitory response. Effect of phase pre-SCI, two-tailed one-way ANOVA, $F_{(3,30)}$ = 0.9, p = 0.454; week 1, two-tailed one-way ANOVA, $F_{(3,30)}$ = 1.18, p = 0.333; week 7, two-tailed Kruskal-Wallis, χ^2 = 0.72, p = 0.868. **c**, boxplots of the percentage of failures to induce changes. Effect of phase pre-SCI, two-tailed one-way ANOVA, $F_{(3,30)}$ = 1.12, p = 0.357; week 1, two tailed Kruskal-Wallis, χ^2 = 4.03, p = 0.258; week 7, two tailed Kruskal-Wallis, χ^2 = 0.49, p = 0.921. **d**, boxplots of MSD response. Effect of phase pre-SCI, two-tailed one-way ANOVA, $F_{(3,30)}$ = 0.21, p = 0.888; week 1, two tailed Kruskal-Wallis, χ^2 = 7.81, p = 0.05; week 7, two-tailed one-way ANOVA, $F_{(3,30)}$ = 0.4, p = 0.753. **e**, graph of plasticity changes and its directionality at week 1 and week 7 for each of the four groups. **f**, color-coded matrices of percentage of mice showing potentiation, no changes, or depression in each group. Sum of each column is 100%. Source data are provided as a Source Data file and on figshare as the dataset "Data and script for EMG response during locomotion (10 ms)" [https://doi.org/10.6084/m9.figshare.c.6925099.v1].

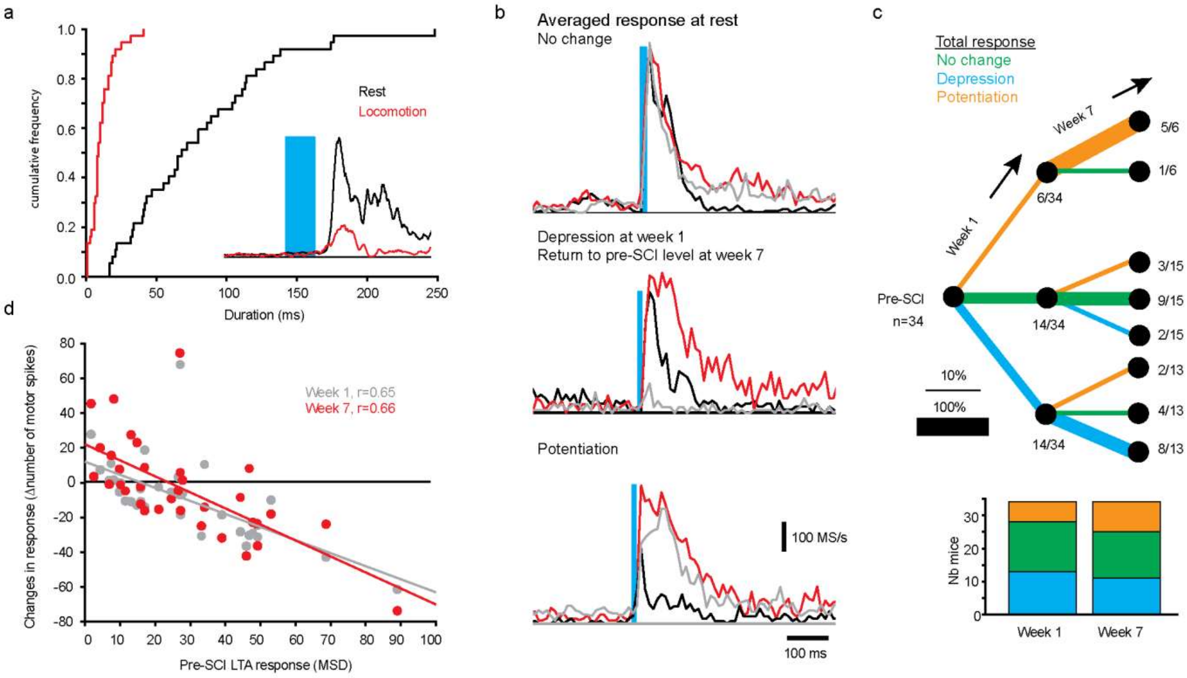

Supplementary Figure 5. Changes of response at rest across medullary reticular groups

a, cumulative distribution function of the duration at rest and during locomotion (n = 34 mice). Paired student t-test, p<0.0001. **b**, examples of PSTH before, one week after, and seven weeks after SCI for each type of plasticity direction. **c**, graph illustrating the direction of plasticity with pooled proportions in the bar graph at the bottom. **d**, scatter plot of changes in response after SCI vs. the response before SCI. Simple linear regression for week 1, $F_{(1,32)}$ = 15.33, p = 0.0004, R^2 = 0.436; at week 7, $F_{(1,32)}$ = 31.59, p < 0.0001, R^2 = 0.422. Source data are provided as a Source Data file.

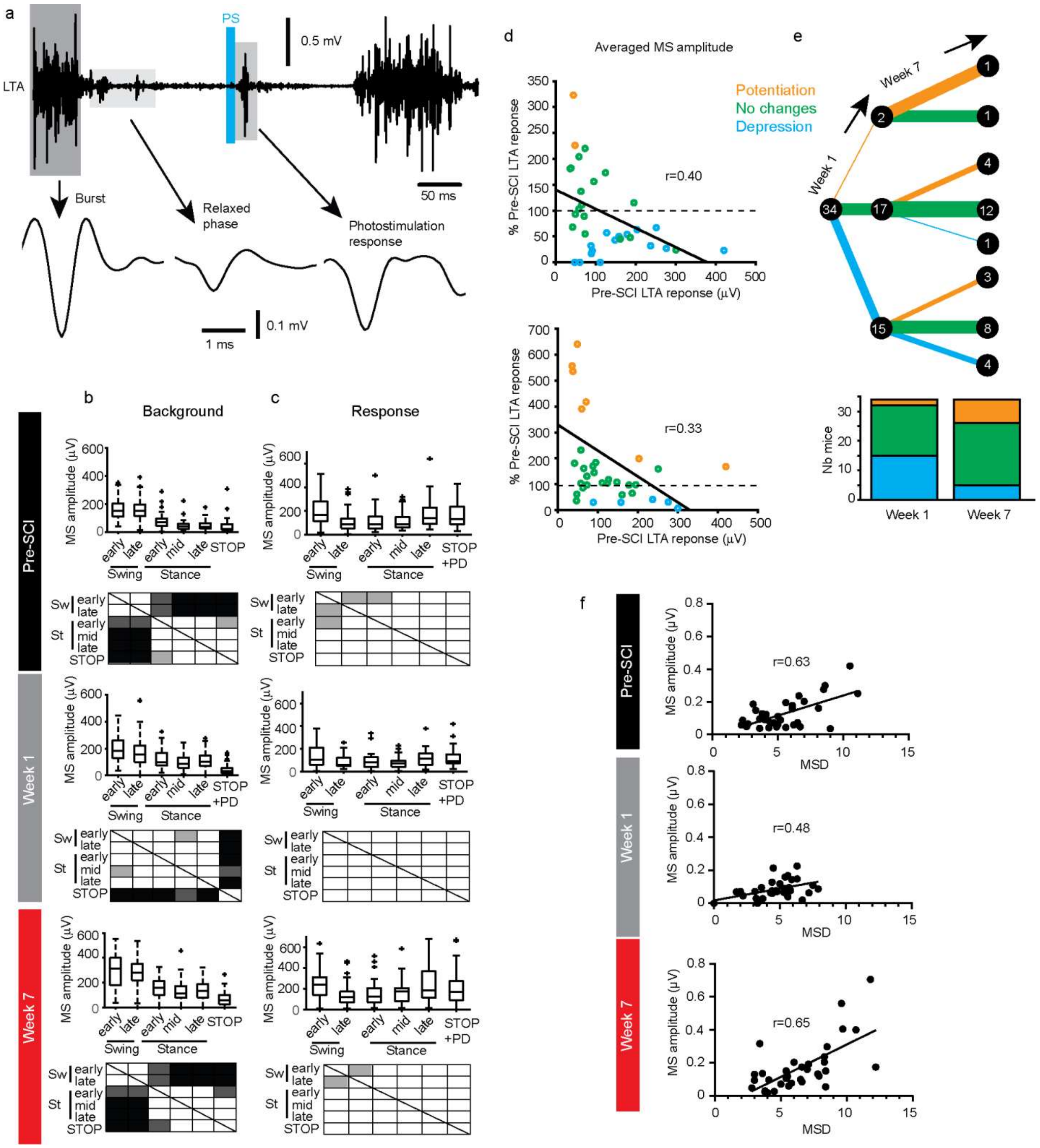

Supplementary Figure 6. EMG response amplitude of the LTA during locomotion

a, example (top) of an LTA EMG response to photostimulation and averaged (bottom) motor spikes during burst, relaxed phase, and in response to photostimulation. **b**, boxplots of motor spike amplitude during background activity. Effect of phase pre-SCI, two-tailed Kruskal-Wallis, χ^2 = 94.3, p < 0.0001; week 1, two-tailed Kruskal-Wallis, χ^2 = 58.5, p < 0.0001; week 7, two-tailed Kruskal-Wallis, χ^2 = 76.6, p < 0.0001. Data from all mice (n = 34). **c**, boxplots of motor

spike response amplitude. Effect of phase pre-SCI, two-tailed Kruskal-Wallis, $\chi^2 = 18.4$, $p = 0.0025$; week 1, two-tailed Kruskal-Wallis, $\chi^2 = 11.8$, $p = 0.0371$; week 7, two-tailed Kruskal-Wallis, $\chi^2 = 14.1$, $p = 0.0152$. **d**, scatter plots of changes in response (% pre-SCI) at week 1 and 7 of the amplitude vs averaged pre-SCI response. Simple linear regression: at week 1, $F_{(1,32)} = 7.495$, $p = 0.01$, $R^2 = 0.164$; at week 7, $F_{(1,32)} = 5.123$, $p = 0.0305$, $R^2 = 0.16$. **e**, graphs illustrating the proportion of mice in each case at week 1 and further changes from week 1 to week 7. Bar graphs at the bottom indicate proportions of each case for the whole sample. **f**, linear regression of amplitude vs. motor spike density upon photostimulation before and after SCI. Simple linear regression: pre-SCI, $F_{(1,32)} = 21.13$, $p < 0.0001$, $R^2 = 0.398$; at week 1, $F_{(1,32)} = 9.777$, $p = 0.0037$, $R^2 = 0.234$; at week 7, $F_{(1,32)} = 24.00$, $p < 0.0001$, $R^2 = 0.429$. Source data are provided as a Source Data file and on figshare as the dataset “Data and script for EMG response during locomotion (10 ms)” [https://doi.org/10.6084/m9.figshare.c.6925099.v1].

Supplementary Table 1. Multiple linear regression of the effect of the lesion extent on the locomotor score, on latency and on plasticity at rest and during locomotion.

	Multiple linear regression		Left side			Right side		
	p-value	R^2	DF	LF	VF	DF	LF	VF
Locomotor score								
Left hindlimb, week 1	0.230	-	-	-	-	-	-	-
Right hindlimb, week 1	0.0009	0.55	-	0.253 (0.004)	-	-	-0.108 (0.003)	-
Left hindlimb, week 7	<0.0001	0.74	-0.07 (0.0111)	0.119 (0.0381)	-	-	-0.109 (<0.0001)	-
Right hindlimb, week 7	<0.0001	0.72	-	0.103 (0.0133)	-	-0.028 (0.0472)	-0.070 (0.0001)	-
Latency								
Week 1	0.003	0.51	-	-	-	-	0.0940 (0.0003)	-
Week 7	0.0108	0.45	-	-	-	-	0.351 (0.0006)	-
Plasticity at rest								
Week 1	0.138	-	-	-	-	-	-	-
Week 7	0.103	-	-	-	-	-	-	-
Plasticity during locomotion								
Week 1	0.046	0.37	-	2.71 (0.0179)	-	-	-	-
Week 7	0.036	0.38	-	-	-	-	-1.4 (0.0212)	-
Horizontal ladder score	0.878		-	-	-	-	-	-

P-values and R2 are shown for the interaction of the extent of lesion of all funiculi on the physiological outcome. Only significant individual slopes for the two-sided multiple linear regressions are shown. P-values are reported below the slope in ( ). DF, dorsal funiculus; LF, lateral funiculus; VF, ventral funiculus.
