# Supplementary material for: Functional plasticity of glutamatergic neurons of medullary reticular nuclei after spinal cord injury in mice
Source: Nat Commun. 2024 Feb 20;15:1542. doi: 10.1038/s41467-024-45300-4 (PMC10879492; doi:10.1038/s41467-024-45300-4)
Supplement: Supplementary file 3 — Reporting Summary [file 41467_2024_45300_MOESM3_ESM.pdf]

nature portfolio

Corresponding author(s): Frédéric Bretzner
Last updated by author(s): Jan 9, 2024

Reporting Summary

Nature Portfolio wishes to improve the reproducibility of the work that we publish. This form provides structure for consistency and transparency in reporting. For further information on Nature Portfolio policies, see our Editorial Policies and the Editorial Policy Checklist.

Statistics

For all statistical analyses, confirm that the following items are present in the figure legend, table legend, main text, or Methods section.

n/a	Confirmed	
☐	☒	The exact sample size (*n*) for each experimental group/condition, given as a discrete number and unit of measurement
☐	☒	A statement on whether measurements were taken from distinct samples or whether the same sample was measured repeatedly
☐	☒	The statistical test(s) used AND whether they are one- or two-sided<br>*Only common tests should be described solely by name; describe more complex techniques in the Methods section.*
☐	☒	A description of all covariates tested
☐	☒	A description of any assumptions or corrections, such as tests of normality and adjustment for multiple comparisons
☐	☒	A full description of the statistical parameters including central tendency (e.g. means) or other basic estimates (e.g. regression coefficient) AND variation (e.g. standard deviation) or associated estimates of uncertainty (e.g. confidence intervals)
☐	☒	For null hypothesis testing, the test statistic (e.g. *F*, *t*, *r*) with confidence intervals, effect sizes, degrees of freedom and *P* value noted<br>*Give P values as exact values whenever suitable.*
☒	☐	For Bayesian analysis, information on the choice of priors and Markov chain Monte Carlo settings
☒	☐	For hierarchical and complex designs, identification of the appropriate level for tests and full reporting of outcomes
☒	☐	Estimates of effect sizes (e.g. Cohen's *d*, Pearson's *r*), indicating how they were calculated

Our web collection on statistics for biologists contains articles on many of the points above.

Software and code

Policy information about availability of computer code

Data collection	Spike2 from CED was used for EMG collection but no code was used. Kinematic data was collected with StreamPix (Norpix)
Data analysis	Kinematic data was analyzed with DeepLabCut. Manual correction were done in Expresso, a custom made software developed by the laboratories of Drs. Serge Rossignol and Trevor Drew. Custom code to analyze EMG and kinematic data were written in Matlab (R2021b) and were deposited on GitHub: https://doi.org/10.5281/zenodo.8206041.

For manuscripts utilizing custom algorithms or software that are central to the research but not yet described in published literature, software must be made available to editors and reviewers. We strongly encourage code deposition in a community repository (e.g. GitHub). See the Nature Portfolio guidelines for submitting code & software for further information.

Data

Policy information about availability of data

All manuscripts must include a data availability statement. This statement should provide the following information, where applicable:

- Accession codes, unique identifiers, or web links for publicly available datasets
- A description of any restrictions on data availability
- For clinical datasets or third party data, please ensure that the statement adheres to our policy

EMG data and coordinates of AAV recombination generated in this study have been deposited in the collection “Data for MRF SCI manuscript” on Figshare [https://doi.org/10.6084/m9.figshare.c.6925099.v1].

Research involving human participants, their data, or biological material

Policy information about studies with human participants or human data. See also policy information about sex, gender (identity/presentation), and sexual orientation and race, ethnicity and racism.

Reporting on sex and gender	N/A
Reporting on race, ethnicity, or other socially relevant groupings	N/A
Population characteristics	N/A
Recruitment	N/A
Ethics oversight	N/A

Note that full information on the approval of the study protocol must also be provided in the manuscript.

Field-specific reporting

Please select the one below that is the best fit for your research. If you are not sure, read the appropriate sections before making your selection.

[x] Life sciences [ ] Behavioural & social sciences [ ] Ecological, evolutionary & environmental sciences

For a reference copy of the document with all sections, see nature.com/documents/nr-reporting-summary-flat.pdf

Life sciences study design

All studies must disclose on these points even when the disclosure is negative.

Sample size	No sample size computation was performed. Changes were statistically assessed on a longitudinal basis for each animal to evaluate the diversity of changes (no changes, depression, potentiation). With a sample of about 7-11 individuals for each of the four lateral positions, between-groups differences could have been detected using ANOVA or Kruskal-Wallis. Also, this sample size per group is usually reported in other locomotor studies, including our owns (Lemieux and Bretzner, 2019; Josset et al., 2018; Roussel et al., 2023).
Data exclusions	Two mouse were excluded after anatomical analysis. Position of implants were deemed too dorsal to be relevant.
Replication	Due to the longitudinal aspect of the study, each mouse is an experiment. Each mouse was sampled at least 4 times: (session) before injury for baseline and after 1, 3 and 7 weeks over a period of 3 months. For each mouse and session, measures were replicated 10-25 times for EMG response at rest (Fig 2), about 30 times for EMG response to 10 ms pulses during locomotion (Fig 3), 4-7 times for kinematic changes during treadmill locomotion (Fig 4), 10-25 times for EMG changes during treadmill locomotion (Fig 5), 10 times for kinematic changes during open field locomotion (Fig 6), 3-5 times for paw placement during treadmill locomotion (Fig 7) and 18-35 times during 3 passages for horizontal ladder locomotion (Fig. 8).
Randomization	Mice were randomly allocated to experimental groups (mediolateral position).
Blinding	Blinding was not possible because experimenters performed both surgeries and data collection. Furthermore, it was possible to judge the group with the lateral position of the implant. All mice underwent the same protocole and data was analyzed the same way.

Reporting for specific materials, systems and methods

We require information from authors about some types of materials, experimental systems and methods used in many studies. Here, indicate whether each material, system or method listed is relevant to your study. If you are not sure if a list item applies to your research, read the appropriate section before selecting a response.

Materials & experimental systems

n/a	Involved in the study	
[x]	[ ]	Antibodies
[x]	[ ]	Eukaryotic cell lines
[x]	[ ]	Palaeontology and archaeology
[ ]	[x]	Animals and other organisms
[x]	[ ]	Clinical data
[x]	[ ]	Dual use research of concern
[x]	[ ]	Plants

Methods

n/a	Involved in the study	
[x]	[ ]	ChIP-seq
[x]	[ ]	Flow cytometry
[x]	[ ]	MRI-based neuroimaging

Animals and other research organisms

Policy information about studies involving animals; ARRIVE guidelines recommended for reporting animal research, and Sex and Gender in Research

Laboratory animals	Mouse: C57/BL6J VGluT2-cre (The Jackson Laboratory, strain 028863) and Ai32 (The Jackson Laboratory, strain 028863)<br>2-3 months old at the start of experimentation.
Wild animals	This study did not involve wild animals
Reporting on sex	This study apply to female only.
Field-collected samples	This study did not involve samples collected from the field
Ethics oversight	Ethical protocol was approved by the local committee for animal protection at Université Laval (Comité de protection des animaux de l'Université Laval, protocol number 19-027) and follow the guidelines of the Canadian Council on Animal Care.

Note that full information on the approval of the study protocol must also be provided in the manuscript.
