# Supplementary material for: Functional plasticity of glutamatergic neurons of medullary reticular nuclei after spinal cord injury in mice
Source: Nat Commun. 2024 Feb 20;15:1542. doi: 10.1038/s41467-024-45300-4 (PMC10879492; doi:10.1038/s41467-024-45300-4)
Supplement: Supplementary file 2 — Peer Review File [file 41467_2024_45300_MOESM2_ESM.docx]

REVIEWER COMMENTS

Reviewer #1 (Remarks to the Author):

In this highly interesting study, Lemieux and colleagues tested the effects of optogenetic stimulation of glutamatergic (Vglut2+) neurons in the medullary reticular formation (MRF) on recovery a few weeks (1-8) after partial unilateral thoracic spinal cord injury in mice. They recorded locomotor output with video cameras or electromyograms. They quantified EMG responses (motor spikes) or used locomotor scores. They found that stimulation sites that evoked weak EMG responses before SCI evoked stronger EMG responses after SCI. Conversely, stimulation sites that evoked strong EMG responses before SCI evoked weaker EMG responses after SCI. They found no lateralization of the motor responses, indicating a strong bilateralization of the stimulation effects. They show that dorsally located Vglut2+ neurons stop or slow down locomotion, whereas the ventrally located ones initiate or speed up locomotion, an interesting observation relative to other recent papers targeting other subtypes of glutamatergic neurons in the MRF. They show that stimulation of Vglut2+ neurons in the MRF improves locomotor score when walking on a ladder. They show that priming Vglut2+ neurons with 5 sessions of tonic stimulation over two weeks improves locomotor score on a ladder. Altogether these results show that targeting Vglut2+ MRF neurons can improve some aspects of locomotor recovery after partial spinal cord injury in mammals.

Strengths. The paper addresses the potential role of a deeply located group of neurons in a brainstem region, the MRF, to improve locomotor function after spinal cord injury. Only a few laboratories in the world can target those regions in vivo. This study comes from the lab of Bretzner, a well-established researcher in the field of locomotor control. His lab has pioneered the characterization of the MLR in mice (Josset et al Curr Biol 2018) and the role of MRF glutamatergic neurons in intact mice (Lemieux et al. 2019 PLoS Biol). His lab has recently demonstrated that the glutamatergic MLR neurons, especially the ones in cuneiform nucleus, are important to recover locomotion after spinal cord injury (Roussel et al. 2023 Cell Rep Med), and here his lab has tested the role of glutamatergic reticular neurons in Gi, Gialpha/GiV, LPGi, and to some extent the intermediate reticular nucleus. The results are clear, and the data, which are already interesting relative to the pathological state, are also very interesting at the fundamental level for the motor control field.

Weaknesses. No major weakness. The experiments were well done and carefully analyzed. The mechanisms involved in the reported effects are not addressed in the study (i.e. why is there such as bilateralization of the effects of the stimulation? Why some sites increase in efficiency and other decrease in efficiency? Why do sessions of tonic priming improve locomotion on the ladder?) but answering these points is beyond the point of the study, and these points are appropriately discussed. Altogether, the study is excellent.

Minor comments. I only have a series of minor comments that should be easy to address to improve slightly the quality of the paper, which is already high.

1. Please be more consistent in the description of the stimulated regions. It is unclear, in many occurrences, what are exactly dorsal MRF and ventral MRF. Maybe define very clearly what these regions are, for instance line 36, using conventional atlas regions (Gi, Giapha/GiV, intermediate nucleus, LPGi).

2. Please report more consistently the number of mice or trials in all legends. It is standard to do so, so that the reader does not need to dig the methods.

3. Please consider improving the abstract. I think it is difficult to intuitively understand it for somebody who has not read the paper.

Details

Abstract:

Line 17-18 “The direction of functional plasticity was anti-correlated with its pre-injury efficacy.”

Please consider clarify this. It is more clear around lines 130-131, when the authors mention that stimulation of sites evoking the strongest muscle responses pre SCI were depressed after SCI, while those evoking the weakest responses got potentiated.

Line 18 “Stimulation”

Please specify “optogenetic stimulation”

Line 19 “dorsal MRF” “ventral sites”

Cf. Minor comment 1.

Line 19 “increased speed”

Please specify before or after SCI

Line 21 “improved stepping ability”

Please specify whether this is on flat ground or during skilled locomotion on ladder.

Line 21 “its conditioning”

Not sure this is the good word. “Conditioning” makes me thing about Pavlovian conditioning. Would “priming”, or simply “sessions of tonic stimulations before the locomotor task” be better to avoid confusion?

Line 44-46 “Dorsal Gi” “ventral Gi”

Cf. Minor comment 1.

Line 56 “of the MRF”

Cf. Minor comment 1.

Line 63 “dorsal MRF (i.e., the Gi)”

Cf. Minor comment 1.

Line 64 “conditioning”

Cf comment about this word.

RESULTS

Line 69

“Given the unilateral projection of glutamatergic reticulospinal neurons”

I think the authors refer to Chx10+ neurons, in the Cregg paper at least, Chx10+ neurons were targeted, not Vglut2+. Please rephrase to be more specific.

Line 89” Given the mainly unilateral projection of glutamatergic reticulospinal pathways of the MRF,”

Please add reference. If the authors are referring to Chx10+ neurons, please replace “glutamatergic” with “Chx10+”. Alternatively, if the authors have in mind a paper showing that Vglut2+ reticulospinal neurons project are mainly ipsilaterally in the spinal cord, please add reference to such paper there.

Line 93 “discrete region”

Left (ipsi lesion) or right or both? Please clarify.

Line 99 “stimulation site”

Medial or lateral contralateral MRF? Or this also includes ipsilesional MRF? Please clarify.

Fig.3F

Please check x axis. Should it be “pre” or “post”?

Line 103

“Changes in reticulospinal efficacy contribute to spontaneous locomotor recovery from SCI”

The fact that these changes are correlated to locomotor recovery does not mean that they contribute to recovery. Please consider modifying this title.

Line 137 “Depression was the next most common observation (12/34)”

Are those conclusions stated based on statistical analyses for each animal or just visual estimation of the signal’s amplitude? Please clarify. If stats were done, please mention those explicitly so that the paper is strengthened.

Line 140, Fig 3f “While there was no correlation between plasticity and locomotor score at week 1, there was one at week 7 (Fig 3h).”

Please provide the significance value in the legend or remove this sentence.

Line 164-175.

Regarding the different behavioral effects of the stimulation sites, would the authors be OK to add a table or a figure summarizing these effects? If yes, I suggest that also include the effects on body turning/head turns that they describe in the next paragraph (line 201-213).

Line 165 “medially and dorsally within the MRF, corresponding to the Gi nucleus”

Cf. Minor comment 1.

Lines 172-175.

Could the authors please state in one sentence around this location whether stimulating ipsi or contralaterally to the lesion had any effect on these behavioral effects post injury?

Lines 209 “turning relying on thoracolumbar segments was reduced”

Please consider specifying what this is.

Lie 2107 “Locomotor deficits”

Please consider specifying which deficits

“line 219-220” “perform misplacement on the dorsum or on the lateral side” “line 221-222 “it was possible to increase the occurrence and the score of correct paw Placements” and “line 223-224 “Taken together, these kinematic changes induced by MRF activation improved stepping ability of chronically impaired SCI mice.”

Considering the importance of this conclusion, is there a bit more more raw data that could be added to illustrate this? Or even simply a short movie?

Line 232 “a conditioning tonic photostimulation”

See comment above about conditioning.

Line 239” improved the global skilled locomotor score for the right” and line 240-241 “the increased probability of successful contact with the rung for the left MRF were comparable to those of the right MRF” and line 244 “to improve functional outcomes after chronic SCI”

Could the authors please specify what exactly the improvement is in a sentence?

Could the authors please specify whether stimulation of the dorsal or ventral MRF was done here?

However, the increased probability of successful contact with the rung for the left MRF were comparable to those of the right MRF (Fig 8e).

Could the authors please specify whether those performances are stronger than those recorded without any stimulation?

Line 260 “correct paw placement of the ipsilesional hindlimb”

I do not remember having a clear distinction between ipsi and contralesional paw placement in the result section. Please check.

Line 261 “conditioning”

See comment above about conditioning.

Line 287 “homeostatic”

Please consider removing this word, not sure the system is trying to reach the same state.

Line 289.” Plasticity of reticulospinal pathways”

The plasticity could also be reticular. Chopek et al. 2021 J Neurophysiol have shown that Chx10+ neurons synapse onto Chx10+ reticulospinal neurons, so maybe consider including this possibility.

Figure legends

Please see Minor comment 2.

Figure 3

MSD

Please define abbreviation.

Line 719 “Significance of the simple linear regression tested with F-test”!

Significance is not given. Please check.

Line 736 “during trains”

Of what? Please specify.

Line 764 “conditioning”

See comment above about conditioning.

Reviewer #2 (Remarks to the Author):

In this paper from Lemieux et al, the authors investigate the role of the glutamatergic reticulospinal tract following lateral thoracic hemisection in mice. The authors demonstrate that glutamatergic drive is disrupted following spinal cord injury independently of the origin, that the functional plasticity is reversely correlated to its pre-injury efficacy, that the dorsal medullary reticular formation decelerate or stop locomotion while the ventral sites increase locomotion speed. Using silencing/stimulation of several specific reticulospinal nuclei via optogenetics they also demonstrate that the activation of descending drive improves stepping ability 7 weeks after SCI while conditioning improved recovery of skilled locomotion. This is a nicely written paper, well-illustrated. The presented data are globally supporting the conclusions and they are of significance for the field. I have the following specific comments to further improve the clarity of the paper:

In general, the conclusions of this paper highly depend on the exact location of the AAVs used for optogenetics and of the exact placement of the optical device. Indeed, most of the reticulospinal nuclei investigated in the paper are extremely small and the results will highly depend on correct placement, injection/spread of the viruses. Therefore, it is crucial to verify 1) the location of the AAV injections and 2) the placement of the optical devices. It is not quite clear whether the map shown in Fig2a has been created from histological sections or from the operative stereotaxic coordinates used to place the optical devices. It is however necessary to create such a map from histological sections and present heatmaps of virus delivery and spread for each animal as well as histological placement of the optical devices for each animal (if this is not what is presented in Fig.2a). If this map has been already generated from histological sections, please mention this in the figure legend as this is currently unclear. I would still suggest presenting the location of the viruses spread as such stimulation/silencing depend on both the virus and the optical device.

Another important point relates to the extend and variability of the lesions in all these animals. How does the lesion location and size influences weight support, locomotor score and the subsequent analysis of the paper? Can the authors generate correlations according to extent and location of the lesions in term of further analysis regarding functional plasticity?

Minor points:

In figure 1a, which kind of staining has been used in this section? There is no mention of any staining in the material and method. How reproducible are the lesions? In figure 1b, what are the “n”?

In general, the “n” are missing in figure legends.

Figure 3,6 and suppl Figures legends: Letters in the figure legend (a,b,c …) are not bolded.

Reviewer #3 (Remarks to the Author):

The manuscript by Lemieux et al. details a very interesting study that builds on the prior work of the group and others. The authors examine the effects of optogenetic stimulation of medullary reticular glutamatergic neurons in intact and spinal cord injured mice to determine functional plasticity that occurs. Surprisingly, ipsilesional (and contralesional) stimulation evoked motor effects of similar latency and magnitude both before and after lateral hemisection. Following SCI, the efficacy of various stimulation sites was equalized compared to pre-injury, with the weakening of stronger connections and the strengthening of weaker connections. This was coincident with locomotor improvements seen with spontaneous recovery of function. Importantly, conditioning stimulation of the MRF improved skilled locomotion in SCI. The findings are significant as they demonstrate plasticity in descending glutamatergic reticulospinal function in short and chronic SCI timepoints, and suggest a potential target for improving locomotion following SCI.

This study contains a lot of valuable data that is difficult to attain and will be of interest within the field and beyond. The methodologies are sound and the data are rigorous. Data transparency was a clear priority of the authors, who made obvious efforts in their displays of individual animal data in all figures. The work largely supports the conclusions, except for a few instances noted below where overstatements should be toned down. The other issues involve the complexity of the data as displayed and described, which make the first half of the Results of the manuscript difficult to follow.

1. The link between locomotor recovery and glutamatergic neuronal functioning is based on a single correlation. Along the same lines, the data demonstrating that stimulation of MRF improves locomotor behavior is not directly compared to spontaneous plasticity in unstimulated mice. Thus, statements asserting causation throughout the text, including the title and line 284-285 should be toned down.

2. The abstract is difficult to make sense of prior to reading the paper. Independent origin, direction of plasticity, and pre-injury efficacy are all references to specific findings in the study that have not yet been explained.

3. The figures are complex. It is really nice that so much of the data is shown in a transparent way. However, given the complexity of the figures and subtle differences between some of them, any mistake in figure labeling and/or reference causes tremendous confusion. In addition to corrections to be made, anything that can be done to help the reader, including edits to the figures (i.e. text at top to differentiate similar figures - “all locomotor cycles” vs “stance and stop”) and sequential ordering (to avoid the jumping back and forth in the supplemental figures referred to out of sequence), would go a long way to the appreciation of the data and the ability to follow the study.

- The title of the upper panel in Figure 2d should be LTA response before SCI.

- Line 99 – Supplementary 2a instead of 2b, I think.

- Suggestion: Move Supplementary 2a to replace current Figure 2b. If current Fig 2a is made to be a bit smaller and possibly reordered, Supp 2b, Fig 2a, c-d would fit within a single figure and take away instance of seemingly unnecessary back and forth, and this would also eliminate one of the examples of subtle differences and partial redundancies that took a few minutes to figure out.

- Supplementary 2c axis should be MSD, not MSN

- Supplementary figures 2 and 3 have numerous errors. The panels do not flow logically (looking at figures only or reading text). The figure legends do not match the figures. There are two Supplementary 2d panels. Additionally, the text references the incorrect panels (lines 116 on – I think the references to Supplementary figs 2d-e should be to Supplementary figs 3d-e), and not all panels are referred to in the text.

- Suggestion – label top of Supplementary 2d and e “All phases of the cycle” to differentiate from Figure 3. Then, Figure 3 e should say “stance and stop” to match the text.

- The data in the top left panels of Supplementary 3b and Supplementary 4 is different but the distinctions between what is displayed in each are not made clear in the text or by the figure legends.

- Supplementary 2b-e are referred to after Supplementary 3 so it should be moved to be afterwards, possibly with Supplementary fig 4 (with clear labels that it is demonstrating ‘rest’ conditions to be compared with Fig 3).

- Line 134 – refers to Supplementary figure 6 but the reason and distinction here was not clear until reaching the conclusion of the section.

- Figure 3 could be clarified by displaying an undisturbed (no photostim) cycle.

4. The correlations in line 85 are there as described but they are not intuitive in all cases. A positive correlation between left LF lesion and recovery is a bit puzzling but may actually fit with recent findings of the Magnuson group (Shepard et al. Silencing long ascending propriospinal neurons after spinal cord injury improves hindlimb stepping in the adult rat. Elife. 2021;10:e70058. doi: 10.7554/eLife.70058.).

5. It is agreed that the finding (in lines 99-101) that access was kept is surprising but more surprising is the lack of change in the latency. This is not plasticity (and not claimed to be) because it is present at week 1. This should be discussed with possible explanations. As it stands, it could be due to tract sparing, despite near complete hemisection, but then one may expect for decreased motor response. It could be circuit redundancy, which would likely lead to different latencies. Another explanation is a difference in the threshold value for stimulation, and therefore different stimulation strengths for pre-SCI, 1 week and 7 weeks. If that is the case, stimulation strengths should be reported because it could offer essential insight into figuring this out.

6. For Figure 6, there should be statistical comparisons for conclusions in lines 208-213. There also appears to be a noticeable increase in the delay post-SCI for both head and body turning.

Minor comments

- Title – Species should be added to the title for clarity.

- Line 36 – First word should be “parts”

- Line 74 – Change the second “evaluate” to a synonym (i.e. determine)

- Line 77 – Missing reference to Figure 1d can be added with 1e.

- Line 108-9: Necessity to swing phase is not shown or explained so should be referenced.

- Line 136: change to “some of those were depressed or potentiated” as it was not the case for all.

- Line 141: The reader should be referred to Supplementary 2b-e (or wherever those panels end up).

- Line 145-8: Please refer to the figures (specific panels) as it is difficult to figure out where this comes from. Part of the issue may be that in line 147, it should be “7/34”, instead of “8/34”, if I followed.

- Lines 164-170: It could be pointed out here that these findings are consistent with prior studies.

- Line 218 - episodes

- Lines 241-242: The statement (“regardless of the plasticity”) should be toned down because only n=2 for depression and it looks different, although it is not statistically significant. If properly powered, it may be.

Reviewer #4 (Remarks to the Author):

I co-reviewed this manuscript with one of the reviewers who provided the listed reports. This is part of the Nature Communications initiative to facilitate training in peer review and to provide appropriate recognition for Early Career Researchers who co-review manuscripts.

**ANSWERS TO REVIEWERS’ COMMENTS**

Reviewer #1 (Remarks to the Author):

In this highly interesting study, Lemieux and colleagues tested the effects of optogenetic stimulation of glutamatergic (Vglut2+) neurons in the medullary reticular formation (MRF) on recovery a few weeks (1-8) after partial unilateral thoracic spinal cord injury in mice. They recorded locomotor output with video cameras or electromyograms. They quantified EMG responses (motor spikes) or used locomotor scores. They found that stimulation sites that evoked weak EMG responses before SCI evoked stronger EMG responses after SCI. Conversely, stimulation sites that evoked strong EMG responses before SCI evoked weaker EMG responses after SCI. They found no lateralization of the motor responses, indicating a strong bilateralization of the stimulation effects. They show that dorsally located Vglut2+ neurons stop or slow down locomotion, whereas the ventrally located ones initiate or speed up locomotion, an interesting observation relative to other recent papers targeting other subtypes of glutamatergic neurons in the MRF. They show that stimulation of Vglut2+ neurons in the MRF improves locomotor score when walking on a ladder. They show that priming Vglut2+ neurons with 5 sessions of tonic stimulation over two weeks improves locomotor score on a ladder. Altogether these results show that targeting Vglut2+ MRF neurons can improve some aspects of locomotor recovery after partial spinal cord injury in mammals.

Strengths. The paper addresses the potential role of a deeply located group of neurons in a brainstem region, the MRF, to improve locomotor function after spinal cord injury. Only a few laboratories in the world can target those regions in vivo. This study comes from the lab of Bretzner, a well-established researcher in the field of locomotor control. His lab has pioneered the characterization of the MLR in mice (Josset et al Curr Biol 2018) and the role of MRF glutamatergic neurons in intact mice (Lemieux et al. 2019 PLoS Biol). His lab has recently demonstrated that the glutamatergic MLR neurons, especially the ones in cuneiform nucleus, are important to recover locomotion after spinal cord injury (Roussel et al. 2023 Cell Rep Med), and here his lab has tested the role of glutamatergic reticular neurons in Gi, Gialpha/GiV, LPGi, and to some extent the intermediate reticular nucleus. The results are clear, and the data, which are already interesting relative to the pathological state, are also very interesting at the fundamental level for the motor control field.

Weaknesses. No major weakness. The experiments were well done and carefully analyzed. The mechanisms involved in the reported effects are not addressed in the study (i.e. why is there such as bilateralization of the effects of the stimulation? Why some sites increase in efficiency and other decrease in efficiency? Why do sessions of tonic priming improve locomotion on the ladder?) but answering these points is beyond the point of the study, and these points are appropriately discussed. Altogether, the study is excellent.

Minor comments. I only have a series of minor comments that should be easy to address to improve slightly the quality of the paper, which is already high.
1. Please be more consistent in the description of the stimulated regions. It is unclear, in many occurrences, what are exactly dorsal MRF and ventral MRF. Maybe define very clearly what these regions are, for instance line 36, using conventional atlas regions (Gi, Giapha/GiV, intermediate nucleus, LPGi).

According to the reviewer’s suggestion, we now used conventional regions instead of dorsal and ventral MRF throughout the text.

2. Please report more consistently the number of mice or trials in all legends. It is standard to do so, so that the reader does not need to dig the methods.

The number of mice and/or trials is now reported in every legend.

3. Please consider improving the abstract. I think it is difficult to intuitively understand it for somebody who has not read the paper.

We modified the abstract to make it easier to read and understand.

Details
Abstract:
Line 17-18 “The direction of functional plasticity was anti-correlated with its pre-injury efficacy.”
Please consider clarify this. It is more clear around lines 130-131, when the authors mention that stimulation of sites evoking the strongest muscle responses pre SCI were depressed after SCI, while those evoking the weakest responses got potentiated.

Thank you for the suggestion, we now use this wording in the abstract.

Line 18 “Stimulation”
Please specify “optogenetic stimulation”

Added.

Line 19 “dorsal MRF” “ventral sites”
Cf. Minor comment 1.

Abstract changes for nuclei nomenclature.

Line 19 “increased speed”
Please specify before or after SCI

We now specify: Before and after SCI, […]

Line 21 “improved stepping ability”
Please specify whether this is on flat ground or during skilled locomotion on ladder.
We now specify both of these: “improved stepping ability on a flat surface of chronically impaired injured mice and its priming improved recovery of skilled locomotion on a horizontal ladder.”

Line 21 “its conditioning”
Not sure this is the good word. “Conditioning” makes me thing about Pavlovian conditioning. Would “priming”, or simply “sessions of tonic stimulations before the locomotor task” be better to avoid confusion?

We agree with the reviewer, we now use the word priming instead of conditioning throughout the manuscript to avoid confusion with Pavlovian conditioning.

Line 44-46 “Dorsal Gi” “ventral Gi”
Cf. Minor comment 1.
We now refer to Gi and GiV.

Line 56 “of the MRF”
Cf. Minor comment 1.

We changed this to: “…of glutamatergic neurons of the Gi, GiV/α, LPGi, and the intermediate reticular nucleus (IRn) to…”

Line 63 “dorsal MRF (i.e., the Gi)”
Cf. Minor comment 1.

“Dorsal” has been removed.

Line 64 “conditioning”
Cf comment about this word.

Changed to priming.

RESULTS
Line 69
“Given the unilateral projection of glutamatergic reticulospinal neurons”
I think the authors refer to Chx10+ neurons, in the Cregg paper at least, Chx10+ neurons were targeted, not Vglut2+. Please rephrase to be more specific.

This sentence has been removed in the new version.

Line 89” Given the mainly unilateral projection of glutamatergic reticulospinal pathways of the MRF,”
Please add reference. If the authors are referring to Chx10+ neurons, please replace “glutamatergic” with “Chx10+”. Alternatively, if the authors have in mind a paper showing that Vglut2+ reticulospinal neurons project are mainly ipsilaterally in the spinal cord, please add reference to such paper there.

Tracing studies in cats, rats, and mice of reticulospinal neurons in general show that there is a significant contralateral component. We have rephrased the sentence to highlight our goal:

“We used a lateral thoracic hemisection as a model to investigate changes in the descending reticulospinal drive to the ipsilesional lumbar network after SCI.”

Line 93 “discrete region”

This has been changed to “spatially restricted (100-200 µm)”

Left (ipsi lesion) or right or both? Please clarify.
We now specify: “…ipsilesional (n = 15 mice) or contralesional (n = 19 mice) MRF…”

Line 99 “stimulation site”
Medial or lateral contralateral MRF? Or this also includes ipsilesional MRF? Please clarify.
We now specify: “…were independent of the medial or lateral position of either ipsi- or contralesional side…”

Fig.3F
Please check x axis. Should it be “pre” or “post”?

It is indeed pre-SCI on the x axis.

Line 103
“Changes in reticulospinal efficacy contribute to spontaneous locomotor recovery from SCI”
The fact that these changes are correlated to locomotor recovery does not mean that they contribute to recovery. Please consider modifying this title.

The title was changed to: “*Changes in reticulospinal efficacy during locomotion after SCI*”.

Line 137 “Depression was the next most common observation (12/34)”
Are those conclusions stated based on statistical analyses for each animal or just visual estimation of the signal’s amplitude? Please clarify. If stats were done, please mention those explicitly so that the paper is strengthened.

We describe this now in an earlier paragraph of the same section: “Using each mouse as its control, we evaluated the direction of plasticity (no changes, depression, or potentiation) after SCI (Fig 3e, Mann-Whitney rank sum test).”

We also added a short paragraph in the Methodology, EMG analysis sub-section, to further describe the approach.

Line 140, Fig 3f “While there was no correlation between plasticity and locomotor score at week 1, there was one at week 7 (Fig 3h).”
Please provide the significance value in the legend or remove this sentence.

p-values were added to the legend for all linear regression.

Line 164-175.
Regarding the different behavioral effects of the stimulation sites, would the authors be OK to add a table or a figure summarizing these effects? If yes, I suggest that also include the effects on body turning/head turns that they describe in the next paragraph (line 201-213).
We have added a Table 1 to summarize the effects on motor behaviors.

Line 165 “medially and dorsally within the MRF, corresponding to the Gi nucleus”
Cf. Minor comment 1.

Changed according to minor comment 1.

Lines 172-175.
Could the authors please state in one sentence around this location whether stimulating ipsi or contralaterally to the lesion had any effect on these behavioral effects post injury?

We modified the last sentence to state this (in bold text): “However, these changes in dynamics triggered by one-second 50 Hz trains were not predicted by the lateralization of the stimulation site (i.e., ipsi- vs. contralesional side, Fig 4c) nor by the plasticity of the locomotor response to shorter (10 ms) pulses (Fig 4d). ”

Lines 209 “turning relying on thoracolumbar segments was reduced”
Please consider specifying what this is.
We have rephrased this: “…body turning, which depends on the coordination of forelimbs, hindlimbs, and axial muscles...”

Lie 2107 “Locomotor deficits”
Please consider specifying which deficits

We have rewritten this sentence to improve clarity: “Despite some recovery (Fig 1), some chronically impaired mice still performed misplacement on the dorsum or on the lateral side of their ipsilesional hindpaw up to seven weeks after SCI (Fig 7a). We tested a sub-group of mice (n = 7 mice) during episode of treadmill locomotion at comfortable steady speed (7-25 cm/s, depending on the mouse).”

“line 219-220” “perform misplacement on the dorsum or on the lateral side” “line 221-222 “it was possible to increase the occurrence and the score of correct paw Placements” and “line 223-224 “Taken together, these kinematic changes induced by MRF activation improved stepping ability of chronically impaired SCI mice.”
Considering the importance of this conclusion, is there a bit more more raw data that could be added to illustrate this? Or even simply a short movie?

We have included Supplementary Movie 1 to illustrate the effects on paw placement.

Line 232 “a conditioning tonic photostimulation”
See comment above about conditioning.

Changed to “priming.”

Line 239” improved the global skilled locomotor score for the right” and line 240-241 “the increased probability of successful contact with the rung for the left MRF were comparable to those of the right MRF” and line 244 “to improve functional outcomes after chronic SCI”

We have new panels d-g to address questions below.

Could the authors please specify what exactly the improvement is in a sentence?

In general, increased score was due to slips at contact being replaced in favor of more partial and correct placements of the paw. In opposition, decreased score was related to more misses and slips (Fig 8f). Almost all mice (n = 5) showing an improvement were associated with potentiation of the locomotor excitatory response, and those with a detrimental effect were associated with depression or no change (Fig 8g).

Could the authors please specify whether stimulation of the dorsal or ventral MRF was done here?

We now provide the map for the effect. Positive effects were observed for the Gi and the LPGi, and detrimental effect for the left intermediate reticular nucleus and GiA/GiV (Fig 8d).

However, the increased probability of successful contact with the rung for the left MRF were comparable to those of the right MRF (Fig 8e).
Could the authors please specify whether those performances are stronger than those recorded without any stimulation?

No changes were observed without priming (Fig 8e).

Line 260 “correct paw placement of the ipsilesional hindlimb”
I do not remember having a clear distinction between ipsi and contralesional paw placement in the result section. Please check.

We now specify this twice in the corresponding Results section (*MRF can improve stepping ability of chronically impaired SCI mice*).

Line 261 “conditioning”
See comment above about conditioning.

Changed to priming.

Line 287 “homeostatic”
Please consider removing this word, not sure the system is trying to reach the same state.

We’d like to keep this concept. This is an interpretation that we have considered along other changes such as neuroanatomical plasticity and changes in intrinsic properties.

Line 289.” Plasticity of reticulospinal pathways”
The plasticity could also be reticular. Chopek et al. 2021 J Neurophysiol have shown that Chx10+ neurons synapse onto Chx10+ reticulospinal neurons, so maybe consider including this possibility.
“Because of a reciprocal connectivity between reticulospinal neurons and reticular interneurons within the medullary reticular formation (Chopek et al., 2021), synaptic and intrinsic plasticity could also occur at the supraspinal level.”

Figure legends
Please see Minor comment 2.

Sample size is now reported in the legend.

Figure 3
MSD
Please define abbreviation.
In the legend: “Boxes indicate time windows for motor spike density (MSD) computation.”

Line 719 “Significance of the simple linear regression tested with F-test”!
Significance is not given. Please check.
We now provide p-values.

Line 736 “during trains”
Of what? Please specify.

Title was changed: “…during trains of photostimulation”

Line 764 “conditioning”
See comment above about conditioning.
Title was changed: “The MRF can be primed to improve skilled locomotion”


Reviewer #2 (Remarks to the Author):

In this paper from Lemieux et al, the authors investigate the role of the glutamatergic reticulospinal tract following lateral thoracic hemisection in mice. The authors demonstrate that glutamatergic drive is disrupted following spinal cord injury independently of the origin, that the functional plasticity is reversely correlated to its pre-injury efficacy, that the dorsal medullary reticular formation decelerate or stop locomotion while the ventral sites increase locomotion speed. Using silencing/stimultion of several specific reticulospinal nuclei via optogenetics they also demonstrate that the activation of descending drive improves stepping ability 7 weeks after SCI while conditioning improved recovery of skilled locomotion. This is a nicely written paper, well-illustrated. The presented data are globally supporting the conclusions and they are of significance for the field. I have the following specific comments to further improve the clarity of the paper:
In general, the conclusions of this paper highly depend on the exact location of the AAVs used for optogenetics and of the exact placement of the optical device. Indeed, most of the reticulospinal nuclei investigated in the paper are extremely small and the results will highly depend on correct placement, injection/spread of the viruses. Therefore, it is crucial to verify 1) the location of the AAV injections and 2) the placement of the optical devices. It is not quite clear whether the map shown in Fig2a has been created from histological sections or from the operative stereotaxic coordinates used to place the optical devices. It is however necessary to create such a map from histological sections and present heatmaps of virus delivery and spread for each animal as well as histological placement of the optical devices for each animal (if this is not what is presented in Fig.2a). If this map has been already generated from histological sections, please mention this in the figure legend as this is currently unclear.

The location of optical implants was derived from histological sections. We have rephrased this in the Histological Analysis section in Methods: “To determine the position of optical implants, the section with the deepest track was imaged for offline measurements of the distance of the center of the tip of the implant from the brainstem midline and from the base of the pyramidal tract.”

Also, in the legend of Figure 2: “a, position of the optical implant evaluated from histological sections of each mouse”

I would still suggest presenting the location of the viruses spread as such stimulation/silencing depend on both the virus and the optical device.

We addressed the viral spread in a new Supplementary Figure 2. Briefly referred to in the Results section, it is described in the Methods section.

“Of the 22 mice that were injected with an AAV-ChR2-eYFP, the signal was found in 20 of them. The signal was very faint for mice injected with AAV-ChR2-mcherry and was therefore not analyzed (n = 4). The eYFP signal was variable from mouse to mouse, but focusing on the eYFP signal we observed a small photobleached region of about 100-200 microns just below the tip. To quantify this (Supplementary Figure 2), we extracted the x-y coordinates of each ChR2-eYFP positive cell and the position of the tip of the optical implant using ImageJ. The distance from these cells to the tip of the optical implant was computed in Matlab for each mouse. The distribution of neurons around the tip for all mice was pooled and shows that this photobleached region of about 100-200 µm matches previous report of bleaching of ChR2-eYFP{Wimmer, 2015 #3417} and GFP signals{Li, 2019 #3414}.”

Another important point relates to the extend and variability of the lesions in all these animals. How does the lesion location and size influences weight support, locomotor score and the subsequent analysis of the paper? Can the authors generate correlations according to extent and location of the lesions in term of further analysis regarding functional plasticity?

Effect of lesion extent is addressed in a new Supplementary Table 1. Specifically, we assessed the impact of lesion size on the locomotor score (former Supplementary Figure 1b-c), latency, plasticity at rest, plasticity during locomotion, and horizontal ladder score.

We mentioned the following points below in the text:

“The level of residual impairment of both hindlimbs seven weeks after SCI could be predicted by the extent of lesion, especially in both lateral funiculi (Supplementary Table 1).”

“Although independent of the stimulation site (Fig 2c), changes in latency were also affected by the lesion size (Supplementary Table 1).”

“While the extent of the lesion size had no influence on the plasticity of motor responses at rest, it influenced excitatory motor responses during locomotion. Indeed, the most extensive lesion on the left side promoted potentiation, whereas the largest lesion on the right side led to depression (Supplementary Table 1).”

“Performance on the horizontal ladder was not influenced by the extent of the lesion as a whole (Supplementary Table 1).”

Minor points:
In figure 1a, which kind of staining has been used in this section? There is no mention of any staining in the material and method.

There was no staining; the dense and the punctate zones of the lesion are auto-fluorescent. This is now mentioned in figure 1 legend.

How reproducible are the lesions? In figure 1b, what are the “n”?

Variability is shown in a new panel b of Figure 1. We report the coefficient of variation to address reproducibility. The former Figure 1b-c has been moved to Supplemental Fig 1 to complement statistical analysis.

“On the left side (ipsilesional side), the lesion extent was maximal in the lateral funiculi and severed about 80% of the ventral and dorsal funiculi (coefficient of variation 12-27%). On the right side, the lesion was more variable (coefficient of variation of 80-200%), usually extending on average to about 32% of the ventral funiculus and 40% of the dorsal funiculus, but it avoided most of the lateral funiculus (Fig 1b). Statistically, the lesion extent was similar for funiculi on the same side and different from those on the other side (Supplemental Fig 1 a-c).”
In general, the “n” are missing in figure legends.

Sample size was added in figure legends when missing.
Figure 3,6 and suppl Figures legends: Letters in the figure legend (a,b,c …) are not bolded.
All letters have been bolded.


Reviewer #3 (Remarks to the Author):

The manuscript by Lemieux et al. details a very interesting study that builds on the prior work of the group and others. The authors examine the effects of optogenetic stimulation of medullary reticular glutamatergic neurons in intact and spinal cord injured mice to determine functional plasticity that occurs. Surprisingly, ipsilesional (and contralesional) stimulation evoked motor effects of similar latency and magnitude both before and after lateral hemisection. Following SCI, the efficacy of various stimulation sites was equalized compared to pre-injury, with the weakening of stronger connections and the strengthening of weaker connections. This was coincident with locomotor improvements seen with spontaneous recovery of function. Importantly, conditioning stimulation of the MRF improved skilled locomotion in SCI. The findings are significant as they demonstrate plasticity in descending glutamatergic reticulospinal function in short and chronic SCI timepoints, and suggest a potential target for improving locomotion following SCI.

This study contains a lot of valuable data that is difficult to attain and will be of interest within the field and beyond. The methodologies are sound and the data are rigorous. Data transparency was a clear priority of the authors, who made obvious efforts in their displays of individual animal data in all figures. The work largely supports the conclusions, except for a few instances noted below where overstatements should be toned down. The other issues involve the complexity of the data as displayed and described, which make the first half of the Results of the manuscript difficult to follow.

1. The link between locomotor recovery and glutamatergic neuronal functioning is based on a single correlation. Along the same lines, the data demonstrating that stimulation of MRF improves locomotor behavior is not directly compared to spontaneous plasticity in unstimulated mice. Thus, statements asserting causation throughout the text, including the title and line 284-285 should be toned down.

We have changed to “contribution by plasticity” to more accurately reflect our study.
The new title is: “Functional plasticity of glutamatergic neurons of medullary reticular nuclei after spinal cord injury in mice”

For line 284-85, we have toned down the causation: “our photostimulation studies show that the descending drive of glutamatergic neurons of the MRF is preserved, albeit with some changes, and could presumably contribute to spontaneous motor recovery after SCI.”

It is has also been changed in other places in the Results section.

2. The abstract is difficult to make sense of prior to reading the paper. Independent origin, direction of plasticity, and pre-injury efficacy are all references to specific findings in the study that have not yet been explained.

In response to suggestions from the reviewers 1 and 3, the abstract has been rewritten to make it more accessible.

3. The figures are complex. It is really nice that so much of the data is shown in a transparent way. However, given the complexity of the figures and subtle differences between some of them, any mistake in figure labeling and/or reference causes tremendous confusion. In addition to corrections to be made, anything that can be done to help the reader, including edits to the figures (i.e. text at top to differentiate similar figures - “all locomotor cycles” vs “stance and stop”) and sequential ordering (to avoid the jumping back and forth in the supplemental figures referred to out of sequence), would go a long way to the appreciation of the data and the ability to follow the study.
- The title of the upper panel in Figure 2d should be LTA response before SCI.

Done.
- Line 99 – Supplementary 2a instead of 2b, I think.
- Suggestion: Move Supplementary 2a to replace current Figure 2b. If current Fig 2a is made to be a bit smaller and possibly reordered, Supp 2b, Fig 2a, c-d would fit within a single figure and take away instance of seemingly unnecessary back and forth, and this would also eliminate one of the examples of subtle differences and partial redundancies that took a few minutes to figure out.

We have made a new Figure 2 by following the reviewer’s advice.
- Supplementary 2c axis should be MSD, not MSN

It was indeed motor spike numbers in comparison to density (per time window of 50 ms) during locomotion. We now avoid abbreviation to avoid confusion.
- Supplementary figures 2 and 3 have numerous errors. The panels do not flow logically (looking at figures only or reading text). The figure legends do not match the figures. There are two Supplementary 2d panels. Additionally, the text references the incorrect panels (lines 116 on – I think the references to Supplementary figs 2d-e should be to Supplementary figs 3d-e), and not all panels are referred to in the text.

It has been corrected.
- Suggestion – label top of Supplementary 2d and e “All phases of the cycle” to differentiate from Figure 3. Then, Figure 3 e should say “stance and stop” to match the text.

Stop was added on top of panel Fig 3e
- The data in the top left panels of Supplementary 3b and Supplementary 4 is different but the distinctions between what is displayed in each are not made clear in the text or by the figure legends.

To avoid diluting data, we removed density amplitudes from Supplementary Fig. 3. With the extra room, averaged data from Supplementary Fig. 4 have been moved to Supplementary Fig. 3. All trials have been discarded as they did not provide much more information.
- Supplementary 2b-e are referred to after Supplementary 3 so it should be moved to be afterwards, possibly with Supplementary fig 4 (with clear labels that it is demonstrating ‘rest’ conditions to be compared with Fig 3).

The figure was moved to Supplemental Fig 5. Label was added.
- Line 134 – refers to Supplementary figure 6 but the reason and distinction here was not clear until reaching the conclusion of the section.

Description of Supplemental Fig 6 now has its own paragraph toward the end of the section. Figure has been changed with amplitude data from previous Supplemental Figures 3 and 4.
- Figure 3 could be clarified by displaying an undisturbed (no photostim) cycle.

We have replaced the example in panel a by an undisturbed cycle.

4. The correlations in line 85 are there as described but they are not intuitive in all cases. A positive correlation between left LF lesion and recovery is a bit puzzling but may actually fit with recent findings of the Magnuson group (Shepard et al. Silencing long ascending propriospinal neurons after spinal cord injury improves hindlimb stepping in the adult rat. Elife. 2021;10:e70058. doi: 10.7554/eLife.70058.).

We agree that it is puzzling, and we were surprised that the regression was significant. Because only a handful of mice seem to be driving this positive correlation in a context of multiple linear regression, we have decided not to discuss this issue. It is possible that a large fraction of spared axons in the lateral funiculus may be deleterious to locomotor performance, which would fit results from Shepard et al. (2021). However, how ascending propriospinal neurons hinder the locomotor score is not more intuitive in the context of our studies. We would thus prefer not to put forward this issue. The initial goal was to show some level of correlation between the lesion and the locomotor score, which is stated in results as such:

“The level of residual impairment of both hindlimbs seven weeks after SCI could be predicted by the extent of lesion, especially in both lateral funiculi (Supplementary Table 1).”

To put less emphasis on the multiple correlation, we have removed the graphical representation from former Supplementary Figure 1b and c and report data instead in a supplementary table.

5. It is agreed that the finding (in lines 99-101) that access was kept is surprising but more surprising is the lack of change in the latency. This is not plasticity (and not claimed to be) because it is present at week 1. This should be discussed with possible explanations. As it stands, it could be due to tract sparing, despite near complete hemisection, but then one may expect for decreased motor response. It could be circuit redundancy, which would likely lead to different latencies. Another explanation is a difference in the threshold value for stimulation, and therefore different stimulation strengths for pre-SCI, 1 week and 7 weeks. If that is the case, stimulation strengths should be reported because it could offer essential insight into figuring this out.

When data were pooled, there was an averaged latency difference between pre-SCI levels and week 1. We included in the results: “The latency was slightly longer at week 1 after SCI (median 18 ms, Wilcoxon signed rank test, p = 0.044) but returned to pre-SCI levels at week 7 (median 16 ms, Wilcoxon signed rank test, p = 0.92). Although independent of the stimulation site (Fig 2c), changes in latency were also affected by the lesion size (Supplementary Table 1). Unexpectedly, changes in motor responses were independent of the stimulation site and lesion size over time after SCI (Fig 2d), thus suggesting that the descending glutamatergic reticulospinal drive kept access to the lumbar spinal circuit below the lesion.”

In the penultimate section in the discussion, we also added*:*

“Interestingly, despite an extensive ipsilateral lesion, ipsilesional motor responses were evoked with a 2ms delay 1 week after SCI, thus suggesting that the cervical propriospinal or contralesional MRF network might relay ipsilesional reticulospinal inputs.”

About threshold, we now mention this in Methods:

“Data reported in this study were obtained at constant laser intensity for the different timepoints.”

6. For Figure 6, there should be statistical comparisons for conclusions in lines 208-213. There also appears to be a noticeable increase in the delay post-SCI for both head and body turning.
Quantification has been added to Figure 6 along with a new Table 1. Previous conclusions were not supported by statistics and were thus changed accordingly. A sentence in the abstract was changed (“The ability to steer was overall preserved”).

Minor comments
- Title – Species should be added to the title for clarity.

Added.
- Line 36 – First word should be “parts”

Done.

- Line 74 – Change the second “evaluate” to a synonym (i.e. determine)

We used determine as suggested.
- Line 77 – Missing reference to Figure 1d can be added with 1e.

Added.
- Line 108-9: Necessity to swing phase is not shown or explained so should be referenced.

It was overstated, we removed it.
- Line 136: change to “some of those were depressed or potentiated” as it was not the case for all.

We have changed it to: “…their responses were either depressed or potentiated at week 7 after SCI in almost two thirds of these mice (9/15).”
- Line 141: The reader should be referred to Supplementary 2b-e (or wherever those panels end up).

The reader is now referred to Supplementary Fig 5a.
- Line 145-8: Please refer to the figures (specific panels) as it is difficult to figure out where this comes from. Part of the issue may be that in line 147, it should be “7/34”, instead of “8/34”, if I followed.

That’s right, there was a mistake (7/34 instead of 8/34). Because the outcome of the Fisher’s test was not significant anymore, we have removed this part.
- Lines 164-170: It could be pointed out here that these findings are consistent with prior studies.

We have included some references: “These results are in line with previous studies (Bouvier et al., 2015; Capelli et al., 2017; Lemieux and Bretzner, 2019; Cregg et al., 2020; Usseglio et al., 2020).”
- Line 218 – episodes

Done.
- Lines 241-242: The statement (“regardless of the plasticity”) should be toned down because only n=2 for depression and it looks different, although it is not statistically significant. If properly powered, it may be.

After reanalyzing data for the priming effect on the horizontal ladder performance for each mouse, we found a relation between plasticity and skilled locomotion improvement.

We state now that “Almost all mice (n = 5/6) showing an improvement were associated with potentiation of their locomotor excitatory responses, whereas those without improvement were associated either with depression or no change (Two-tailed Fisher’s exact test, p = 0.043, Fig 8g).”

**REVIEWERS' COMMENTS**

Reviewer #1 (Remarks to the Author):

The authors perfectly addressed all my concerns.

Reviewer #2 (Remarks to the Author):

The paper is now improved. All my comments have been answered and my suggestions implemented.

Reviewer #3 (Remarks to the Author):

We are satisfied by the authors' responses to the prior review and the corresponding improvements to the paper. We have no further critiques.

Reviewer #4 (Remarks to the Author):

I co-reviewed this manuscript with one of the reviewers who provided the listed reports. This is part of the Nature Communications initiative to facilitate training in peer review and to provide appropriate recognition for Early Career Researchers who co-review manuscripts
